# Supplementary material for: Autophagy regulates fatty acid availability for oxidative phosphorylation through mitochondria-endoplasmic reticulum contact sites
Source: Nat Commun. 2020 Aug 13;11:4056. doi: 10.1038/s41467-020-17882-2 (PMC7426880; doi:10.1038/s41467-020-17882-2)
Supplement: Supplementary file 1 — Supplementary information [file 41467_2020_17882_MOESM1_ESM.pdf]

Supplementary Information

Autophagy regulates fatty acid availability for oxidative phosphorylation through mitochondria-endoplasmic reticulum contact sites

Bosc*, Broin* et *al*.

Table of Contents :

- **Supplementary Tables 1 - 5**
- **Supplementary Figures 1-8**

Supplementary Tables

	ID study	Sexe	Karyotype	Cytogenetic risk group	FAB	FLT3 statut	IDH statut		NPM1 statut	DNMT3A statut	CEBPα statut	P53 statut
							IDH1	IDH2				
		(M=1, F=2)		Infavorable: 0<br>Intermediate: 1<br>Favorable: 2		(ITD 0/1)	0/1	0/1	0/1	0/1	0/1	0/1
Primary cells	**LAI**	1	47,XY,inv(16)(p13q22),+22<15>/46,XY<1>	2	4	0	0	0	0	0	0	ND
	**GAR**	1	47,XY,+8<11>/46,XY<9>	1	4	0	ND	ND	0	ND	0	ND
	**MAR**	2	46,XX<20>	1	1	0	0	0	1	1	0	ND
	**MALO**	2	46,XX<20>	1	1	1	0	0	1	1	0	0
	**FEG**	1	47,XY,inv(16)(p13q22),+22<20>	2	4	ND	ND	ND	ND	ND	ND	ND
	**AUB**	1	46,XY<20>	1	4	1	0	0	1	1	0	ND
	**AUB**	1	46,XY<20>	1	1	1	0	0	1	1	0	0
	**LOP**	1	46,XX<20>			1	ND	ND	0	0	ND	ND
	**ZEG**	2	*46,XX,del(5)(p15p13),add(6)(q2?5),add(14)(q3?2)<17>/46,XX<4>*	0	1	1	ND	ND	0	ND	ND	ND
	**LAMA**	2	46,XX,der(5)t(5;14)(q13;q12),der(14)t(5;14)(q15;q12)[2]/46,XX[18]									
	**LOP**	1	47,XY,+8,inv(16)(p13q22)<20>	2								
	**SAGE**	2	46,XX<20>	1	5	0	0	0	1	1	0	0
	**ELLUL**	1	46,XY<20>	1	4	1	0	0	0	0	0	0
	**BOMA**	2	Echec	NA	2	1	0	0	1	ND	ND	ND
	**DELA**	2	46,XX<20>	1	4	1	0	0	1	0	0	0
	**BES**	2	t(9;22)	0	6	0	0	0	1	0	0	ND
	**PRA**	2	complex	0	2	0	0	0	0	0	0	1
	**FRE**	1	46,XY<20>	1	5	0	1	0	1	1	0	ND
	**LAS**	1	46,XY<20>	1	2	0	0	0	1	0	0	ND
	**BOUMA**	2	46,XX<20>	1	4	1	ND	ND	1	ND	ND	ND
Cell lines	**MOLM14**	1	complex		5	1	0	0	0	0	0	0
	**U937**	1	t(10;11)(p13;q14)		5	0	0	0	0	0	0	1

Supplementary Table 1. Primary and AML cell line characteristics

Sample type	From	Birth date	Immatriculation	Sexe	Cell isolation
**Cord blood**	EFS, Besançon	24/09/2019	64195204962	M	CD34+
**Cord blood**	EFS, Besançon	23/09/2019	64195204989	F	CD34+
**Cord blood**	EFS, Besançon	04/11/2019	6419550633 8	M	CD34+
**Cord blood**	EFS, Besançon	04/11/2019	6419520582 6	F	CD34+
**Cord blood**	EFS, Besançon	25/11/2019	6419550675 -	F	CD34+
**Cord blood**	EFS, Besançon	25/11/2019	6419550627 4	M	CD34+
**Cord blood**	EFS, Besançon	02/12/2019	6419530546 4	F	CD34+
**Cord blood**	EFS, Besançon	02/12/2019	6419550684 8	M	CD34+
**Cord blood**	EFS, Besançon	10/12/2019	6419550697 9	M	CD34+
**Cord blood**	EFS, Besançon	10/12/2019	6419520647 4	M	CD34+
**Cord blood**	EFS, Besançon	17/12/2019	6419550712 -	F	CD34+
**Cord blood**	EFS, Besançon	16/12/2019	6419520662 6	F	CD34+
**Peripheral blood**					
11 samples	EFS, Toulouse				PBMC

Supplementary Table 2. Healthy donor characteristics

MOLM14 METF vs CTL UP			MOLM14 METF VS CTL DOWN			U937 METF VS CTL UP			U937 METF VS CTL DOWN		
Genes	FC	p value	Genes	FC	p value	Genes	FC	p value	Genes	FC	p value
RRP7B	2.5	0.037	TBC1D30	0.27	0	LOC284561	5.21	0.001	INSIG1	0.23	0
ULBP1	2.4	0.005	TXNIP	0.32	0.007	SLC7A11	4.1	0.029	SNAR-E	0.25	0
CTH	2.39	0	VNN1	0.4	0.006	SESN2	3.57	0.005	LDLR	0.31	0.007
JHDM1D	2.25	0.001	MPO	0.4	0.001	DDIT4	3.29	0.002	IL8	0.32	0.002
IL31RA	2.17	0.006	LOC100505613	0.42	0.004	TUBE1	3.13	0	TARP	0.38	0
ATF5	2.16	0.004	MNDA	0.43	0.015	MIR628	3.05	0.049	SNAR-B1	0.39	0.001
TUBE1	2.14	0	RN5S141	0.43	0	DDIT3	3.03	0	MSMO1	0.41	0.001
SLC6A9	2.05	0.006	INSIG1	0.45	0.003	LOC374443	2.79	0	SDF2L1	0.41	0.002
TRIB3	1.92	0.001	TMEM45A	0.45	0.02	RRP7B	2.79	0.002	HSPA1B	0.41	0
MIR153-2	1.86	0.002	MYBPH	0.46	0.006	CTH	2.53	0.003	MIR3676	0.41	0.007
FLJ46906	1.85	0.001	TARP	0.46	0.001	ULBP1	2.49	0.046	GPR84	0.42	0.005
ME1	1.81	0.001	ADAMTS1	0.48	0.003	IL31RA	2.42	0.008	HSPA1A	0.42	0.006
P2RX7	1.81	0.008	ANXA1	0.48	0.001	ZC3H6	2.4	0.013	RAB39A	0.44	0.035
GNG7	1.8	0.001	STS	0.49	0	P2RX7	2.18	0.006	IPCEF1	0.44	0
ZNF643	1.79	0.045	HIST1H2AB	0.5	0.024	ASNS	2.15	0.005	FASN	0.45	0.006
RASA4	1.78	0.056	LOC642934	0.5	0.016	CAB39L	2.1	0.007	SPRR2F	0.45	0.004
FLJ35024	1.77	0.001	CHCHD7	0.51	0.003	PSAT1	2.1	0.002	ECRP	0.46	0.002
IGANRP	1.74	0.022	HIST1H2BG	0.52	0.01	HCK	2.07	0.004	DHCR24	0.46	0.002
SERPINI2	1.73	0.013	E2F8	0.54	0.003	TSEN15	2.02	0.002	SNORD14E	0.46	0.014
C5orf28	1.72	0.001	CCR2	0.55	0.019	CCDC146	1.96	0.023	SNAR-D	0.47	0.025
HEATR5A	1.71	0.012	PCED1B-AS1	0.55	0.005	WARS	1.92	0.006	HSPA8	0.48	0.002
SLC7A11	1.71	0.003	FADS1	0.57	0.038	FBXO10	1.92	0.001	HMGCS1	0.48	0
LOC10050696	1.7	0.019	LOC100506394	0.57	0.007	SPG11	1.91	0.01	DNAJA1	0.48	0.001
NHSL1	1.69	0.009	IRS2	0.57	0.004	VLDLR	1.87	0.002	CCL2	0.49	0.002
LOC10012828	1.67	0.01	E2F7	0.57	0.007	ERN1	1.84	0.003	RNU12	0.5	0.001
CCDC146	1.66	0.001	MIR181B1	0.57	0.006	GARS	1.84	0.003	HMGCR	0.5	0
DDIT4	1.66	0.003	C19orf59	0.58	0.001	AARS	1.84	0	POP1	0.51	0.004
MIR628	1.66	0.002	GDAP1	0.58	0.006	SOS2	1.83	0.001	MIR4521	0.51	0.04
LOC10013015	1.65	0.015	HIST1H3J	0.58	0.004	CLIC4	1.81	0	CYB5D1	0.52	0.035
SNORD96B	1.64	0.009	GPR183	0.58	0	LOC389765	1.81	0.009	SNAR-C1	0.52	0.001
SARS	1.64	0.002	TK1	0.59	0.004	FRA10AC1	1.81	0.034	CYP51A1	0.52	0
SESN2	1.64	0.007	DHCR24	0.6	0	EDEM2	1.8	0.029	ANKRD22	0.53	0
PPP1R11	1.62	0.031	SLPI	0.6	0.007	PDCD4	1.78	0.009	MIR221	0.53	0.003
PSAT1	1.62	0	GINS2	0.6	0.001	VEGFA	1.78	0	TSR2	0.53	0.011
VEGFA	1.61	0.001	FCGR2B	0.61	0.027	ANKRA2	1.78	0.036	CCNE2	0.54	0.01
PAG1	1.61	0.029	NAV3	0.61	0.008	DNAJC5B	1.78	0.001	SQLE	0.54	0.001
DDIT3	1.6	0.001	HSD11B1	0.61	0	HPS4	1.77	0.001	CLEC4D	0.55	0.034
STC2	1.6	0.02	KIF20A	0.62	0.002	PLK1S1	1.76	0	SNAR-H	0.55	0.002
FBXL20	1.59	0.004	CABLES2	0.62	0.024	RPS6KA2-IT1	1.76	0.001	STRA13	0.55	0.016
CYP2B7P1	1.59	0.007	SDF2L1	0.63	0.03	RPS6KA2	1.76	0.005	STARD4	0.55	0.013
RN5S465	1.57	0.001	ARRB2	0.63	0	ACACB	1.76	0.005	SF3B5	0.55	0
TMEM63A	1.56	0.004	ACPP	0.63	0.01	JHDM1D	1.75	0.012	GPRIN3	0.55	0.017
C5orf56	1.56	0.026	CSF1R	0.63	0.005	CTSO	1.75	0.043	LCMT2	0.55	0.044
CRYM-AS1	1.55	0.015	HSD17B7	0.64	0.007	HSD17B13	1.74	0.006	JUN	0.56	0.024
PLXNA1	1.55	0.033	MS4A3	0.64	0.002	SENP7	1.73	0.037	PCNA-AS1	0.56	0.003
SYCP2	1.55	0.017	NAB2	0.64	0.045	VAMP4	1.73	0.012	HSPH1	0.56	0
ASNS	1.54	0	S100A8	0.64	0.002	DNASE2	1.72	0.019	TRGV10	0.56	0.01
IFIH1	1.54	0.011	LDLR	0.64	0.023	ZMAT1	1.71	0.013	CCNE1	0.56	0.001
ALDH1L2	1.54	0	PCED1B	0.64	0.001	ITGAL	1.71	0.001	TNFRSF12A	0.57	0.028
LRP5	1.54	0.001	PGBD5	0.64	0.021	KCNE1	1.71	0.002	MFSD12	0.57	0.028
IRF1	1.54	0	RRM2	0.64	0.001	TNFRSF14	1.69	0.006	GINS2	0.57	0
LOC10050667	1.54	0.011	HIST1H2BM	0.64	0.017	LOC10050700	1.69	0.045	TREM1	0.57	0.004
ZP3	1.54	0.005	FOXA1	0.64	0.014	MARS	1.69	0.003	IDI1	0.57	0.003
ZC3H6	1.53	0.001	CCNA1	0.64	0.014	MGEA5	1.69	0.008	TNF	0.58	0.003
UBA7	1.53	0.011	C6orf223	0.64	0.007	NHS	1.69	0.013	KBTBD8	0.58	0.009
ANK2	1.53	0.004	GAPT	0.65	0.017	C6orf48	1.68	0	HLA-DQB1-AS1	0.58	0.044
C12orf66	1.53	0.001	CD300A	0.65	0.017	LRRC2	1.68	0.012	PANX1	0.58	0.005
ANKRD16	1.53	0.015	GBP2	0.65	0.003	FAM214A	1.68	0.004	CEACAM6	0.58	0.042
LOC10050573	1.53	0.031	PLAG1	0.65	0.017	GDAP1L1	1.67	0.004	MLKL	0.58	0.009
RRN3P2	1.53	0.002	PCNA-AS1	0.65	0.048	MIR580	1.67	0.031	GRWD1	0.58	0.018
GUSBP4	1.53	0.016	SPN	0.65	0.008	HOXA2	1.67	0.013	MOCS3	0.58	0
USP32P2	1.53	0.029	KIF4A	0.65	0.006	SPNS3	1.66	0	DHRS9	0.58	0.018
BBS2	1.52	0	RGS18	0.66	0.018	TRIB3	1.66	0	RN5S53	0.58	0.012
PVT1	1.52	0.047	GABRA4	0.66	0.006	SETDB2	1.66	0.004	DANCR	0.58	0.003
LOC10050668	1.52	0.015	LSS	0.66	0.002	SYTL1	1.65	0.001	LOC100288432	0.59	0
SNX29P2	1.52	0.016	TRAJ17	0.67	0.006	AOAH	1.65	0.004	IL22RA2	0.59	0.024
LOC728190	1.51	0.004	NFE2	0.67	0.019	PCK2	1.65	0.003	E2F2	0.59	0.003
MSS51	1.51	0.001	CD83	0.67	0.024	ASAH1	1.64	0.002	DCTPP1	0.59	0.007
ZNF354B	1.51	0.003	SOCS1	0.67	0.007	CNTNAP3	1.64	0.013	ALDH1B1	0.59	0.005
LOC10065284	1.51	0.048	SLC46A3	0.67	0.018	TANC2	1.64	0.008	GPR183	0.59	0.01
WHAMMP2	1.5	0.023	RN5S335	0.67	0.026	LYRM5	1.64	0.017	FBXO33	0.59	0.007
SLC22A15	1.5	0.018	PDK3	0.67	0.038	HLCS	1.64	0.018	C1orf38	0.6	0.002
RASA4B	1.5	0.004	ACAT2	0.68	0.006	NHSL1	1.64	0.001	FAM57A	0.6	0.004
RN5S474	1.49	0.012	BNIP3	0.68	0.002	CARS	1.64	0	C19orf10	0.6	0.015
SOCS7	1.49	0.006	TNFRSF19	0.68	0.016	FAM129A	1.63	0.01	TOE1	0.6	0.008
LOC10019098	1.49	0.029	METTL7A	0.68	0.016	RNY4P1	1.63	0.023	HNRNPM	0.6	0.002
ADAT2	1.49	0.005	DEPDC7	0.68	0.025	MANBA	1.63	0.019	CDC6	0.6	0.002
GPCPD1	1.49	0	STRA13	0.68	0.001	PHKB	1.63	0.022	LOC100130698	0.6	0.016
LENG8-AS1	1.49	0	HIST1H4D	0.68	0.031	ARMC12	1.63	0	HIST1H3I	0.61	0.005
LOC10050676	1.48	0.002	CD36	0.68	0.029	ST6GALNAC3	1.63	0.01	CD180	0.61	0.005
FLJ13197	1.48	0.024	LOC100653350	0.68	0.003	OSCAR	1.62	0.024	RNU5E-4P	0.61	0.014
LOC10050732	1.48	0.011	POU3F2	0.68	0.023	UBXN2B	1.62	0.001	MRPS12	0.61	0.002
PARP6	1.48	0.026	IGHM	0.68	0.039	CENPJ	1.62	0.002	MEX3D	0.61	0.002
ZFP62	1.48	0	IPCEF1	0.68	0.005	CNTNAP3B	1.62	0.022	MCAT	0.62	0.011
LONP1	1.48	0.001	KLF6	0.68	0.013	ARSD	1.61	0.001	MMP8	0.62	0.049
H6PD	1.47	0.048	NEFH	0.68	0.006	SARS	1.61	0.031	PCYT1B	0.62	0.004
LOC728888	1.47	0.005	RNASE3	0.69	0.003	C5AR1	1.6	0.032	LRRC59	0.62	0.002
TLR2	1.47	0.011	HMGCS1	0.69	0.009	RNASEH2B	1.6	0.008	CCDC86	0.62	0.003
UTS2	1.47	0.003	FBXO33	0.69	0.03	ZNF506	1.6	0.022	RRS1	0.62	0.017
DPY19L2P3	1.47	0.004	TLCD1	0.69	0.034	TAAR5	1.6	0.008	UTP3	0.62	0.003
CUL9	1.47	0.01	POLA1	0.69	0.022	ERMP1	1.6	0.003	MIR3661	0.62	0.045
PLD6	1.47	0.003	HIST1H2BL	0.69	0.01	ERAP2	1.59	0.014	PGAM1	0.62	0
FAM91A2	1.46	0.004	FASN	0.69	0.001	TMEM9B-AS1	1.59	0.006	NXPE3	0.62	0.008
D2HGDH	1.46	0.041	ELAVL4	0.69	0.009	XPOT	1.59	0.003	SSSCA1	0.63	0.022
PLXNC1	1.46	0.006	LINC00467	0.69	0.008	SKIL	1.58	0.001	DHCR7	0.63	0.018
IL15RA	1.46	0.007	CX3CR1	0.69	0.001	C2	1.58	0.033	C3AR1	0.63	0.017
GSDMB	1.46	0.004	DLGAP5	0.69	0.021	FIG4	1.57	0.032	RNU5A-8P	0.63	0.026
FLJ45340	1.46	0.015	GBE1	0.69	0.003	FAM70B	1.57	0.001	CD3EAP	0.63	0.008
LOC10050673	1.46	0.007	BNIP3L	0.69	0.017	C6orf165	1.57	0.026	UHRF1	0.63	0.003
BTN2A3P	1.45	0.005	SPR	0.7	0.006	MIR548I4	1.57	0.049	POLR2L	0.63	0.013

Supplementary Table 3. Top 100 differentially expressed genes for MOLM14 and U937 metformin vs control.
Transcriptome profiling assays on MOLM14 and U937 treated or not with 10mM metformin in independent triplicates (1 experiment). GEO: GSE97346.

MOLM14/U937 COMMON UP ; #33	MOLM14/U937 COMMON DOWN ; #28
CTH	PCNA-AS1
PSAT1	FADS1
TUBE1	HIST1H4D
ASNS	SDF2L1
SPG11	FBXO33
BBS2	HIST1H2AB
RASA4	CD83
JHDM1D	CABLES2
ZC3H6	LDLR
TRIB3	GAPT
VEGFA	MSMO1
DDIT3	CCNA1
SARS	HMGCS1
MIR628	ACAT2
DDIT4	GDAP1
SLC7A11	TRAJ17
ATF5	IPCEF1
ADAT2	INSIG1
ULBP1	E2F8
IL31RA	RNASE3
SESN2	GINS2
BACE1-AS	FASN
P2RX7	ANXA1
NHSL1	TARP
RASA4	RRM2
CUL9	STRA13
TAP2	GPR183
TAP2	DHCR24
HLCS	
SKIL	
RASA4	
PCK2	
RRP7B	

Supplementary Table 4. MOLM14 and U937 common UP and DOWN genes for metformin vs control.
Transcriptome profiling assays on MOLM14 and U937 treated or not with 10mM metformin in independent triplicates (1 experiment). GEO: GSE97346.

	GO-Term	GO-Term id	P-value	List of observed genes
COMMON DOWN-regulated	organic substance biosynthetic process	GO:1901576	0.01	FADS1, DHCR24, LDLR, FASN, RRM2, HIST1H4D, ANXA1, HMGCS1, ACAT2, GINS2, INSIG1, MSMO1, CCNA1, E2F8
	small molecule metabolic process	GO:0044281	0	FADS1, DHCR24, LDLR, FASN, RRM2, ANXA1, HMGCS1, ACAT2, INSIG1, MSMO1
	**lipid metabolic process**	**GO:0006629**	**0**	**FADS1, DHCR24, LDLR, FASN, ANXA1, HMGCS1, ACAT2, INSIG1, MSMO1**
	small molecule biosynthetic process	GO:0044283	0	FADS1, DHCR24, FASN, ANXA1, HMGCS1, ACAT2, INSIG1, MSMO1
	cellular lipid metabolic process	GO:0044255	0	FADS1, LDLR, FASN, ANXA1, HMGCS1, ACAT2, INSIG1, MSMO1
	cholesterol metabolic process	GO:0008203	0	DHCR24, LDLR, FASN, HMGCS1, ACAT2, INSIG1, MSMO1
	secondary alcohol metabolic process	GO:1902652	0	DHCR24, LDLR, FASN, HMGCS1, ACAT2, INSIG1, MSMO1
	sterol metabolic process	GO:0016125	0	DHCR24, LDLR, FASN, HMGCS1, ACAT2, INSIG1, MSMO1
	steroid metabolic process	GO:0008202	0	DHCR24, LDLR, FASN, HMGCS1, ACAT2, INSIG1, MSMO1
	alcohol metabolic process	GO:0006066	0	DHCR24, LDLR, FASN, HMGCS1, ACAT2, INSIG1, MSMO1
	organic hydroxy compound metabolic process	GO:1901615	0	DHCR24, LDLR, FASN, HMGCS1, ACAT2, INSIG1, MSMO1
	**regulation of cell differentiation**	**GO:0045595**	**0.01**	**FADS1, LDLR, HIST1H4D, ANXA1, CD83, GPR183, INSIG1**
	**cellular response to stress**	**GO:0033554**	**0.01**	**FADS1, SDF2L1, HIST1H4D, ANXA1, GINS2, INSIG1, E2F8**
	cholesterol biosynthetic process	GO:0006695	0	DHCR24, FASN, HMGCS1, ACAT2, INSIG1, MSMO1
	secondary alcohol biosynthetic process	GO:1902653	0	DHCR24, FASN, HMGCS1, ACAT2, INSIG1, MSMO1
	sterol biosynthetic process	GO:0016126	0	DHCR24, FASN, HMGCS1, ACAT2, INSIG1, MSMO1
	alcohol biosynthetic process	GO:0046165	0	DHCR24, FASN, HMGCS1, ACAT2, INSIG1, MSMO1
	steroid biosynthetic process	GO:0006694	0	DHCR24, FASN, HMGCS1, ACAT2, INSIG1, MSMO1
	organic hydroxy compound biosynthetic process	GO:1901617	0	DHCR24, FASN, HMGCS1, ACAT2, INSIG1, MSMO1
	fatty acid metabolic process	GO:0006631	0	FADS1, FASN, ANXA1, ACAT2, INSIG1, MSMO1
	regulation of lipid metabolic process	GO:0019216	0	FADS1, LDLR, FASN, ANXA1, HMGCS1, INSIG1
	monocarboxylic acid metabolic process	GO:0032787	0	FADS1, FASN, ANXA1, ACAT2, INSIG1, MSMO1
	oxidation-reduction process	GO:0055114	0	FADS1, DHCR24, FASN, RRM2, ACAT2, MSMO1
	carboxylic acid metabolic process	GO:0019752	0	FADS1, FASN, ANXA1, ACAT2, INSIG1, MSMO1
	oxoacid metabolic process	GO:0043436	0	FADS1, FASN, ANXA1, ACAT2, INSIG1, MSMO1
	organic acid metabolic process	GO:0006082	0	FADS1, FASN, ANXA1, ACAT2, INSIG1, MSMO1
	**regulation of cell cycle**	**GO:0051726**	**0**	**DHCR24, RRM2, ANXA1, CCNA1, CABLES2, E2F8**
	leukocyte activation	GO:0045321	0	RNASE3, LDLR, GAPT, ANXA1, CD83, GPR183
	cell activation	GO:0001775	0.01	RNASE3, LDLR, GAPT, ANXA1, CD83, GPR183
	regulation of lipid biosynthetic process	GO:0046890	0	LDLR, FASN, ANXA1, HMGCS1, INSIG1
	regulation of small molecule metabolic process	GO:0062012	0	LDLR, FASN, ANXA1, HMGCS1, INSIG1
	cellular response to lipid	GO:0071396	0	GDAP1, LDLR, ANXA1, HMGCS1, INSIG1
	hemopoiesis	GO:0030097	0	FASN, HIST1H4D, ANXA1, CD83, GPR183
	hematopoietic or lymphoid organ development	GO:0048534	0	FASN, HIST1H4D, ANXA1, CD83, GPR183
	response to lipid	GO:0033993	0.01	GDAP1, LDLR, ANXA1, HMGCS1, INSIG1
	response to organic cyclic compound	GO:0014070	0.01	GDAP1, ANXA1, CD83, HMGCS1, INSIG1
	immune system development	GO:0002520	0.01	FASN, HIST1H4D, ANXA1, CD83, GPR183
	regulation of steroid metabolic process	GO:0019218	0	LDLR, FASN, HMGCS1, INSIG1
	fatty acid biosynthetic process	GO:0006633	0	FADS1, FASN, ANXA1, INSIG1
	G1/S transition of mitotic cell cycle	GO:0000082	0	RRM2, ANXA1, CCNA1, E2F8
	cell cycle G1/S phase transition	GO:0044843	0	RRM2, ANXA1, CCNA1, E2F8
	monocarboxylic acid biosynthetic process	GO:0072330	0	FADS1, FASN, ANXA1, INSIG1
	carboxylic acid biosynthetic process	GO:0046394	0	FADS1, FASN, ANXA1, INSIG1
	organic acid biosynthetic process	GO:0016053	0	FADS1, FASN, ANXA1, INSIG1
	gland development	GO:0048732	0	FASN, ANXA1, HMGCS1, E2F8
	response to nutrient levels	GO:0031667	0	FADS1, GDAP1, LDLR, HMGCS1
	leukocyte differentiation	GO:0002521	0	FASN, ANXA1, CD83, GPR183
	response to extracellular stimulus	GO:0009991	0	FADS1, GDAP1, LDLR, HMGCS1
	mitotic cell cycle phase transition	GO:0044772	0	RRM2, ANXA1, CCNA1, E2F8
	cellular response to organic cyclic compound	GO:0071407	0	GDAP1, ANXA1, HMGCS1, INSIG1
	regulation of leukocyte activation	GO:0002694	0.01	LDLR, ANXA1, CD83, GPR183
	cell cycle phase transition	GO:0044770	0.01	RRM2, ANXA1, CCNA1, E2F8
	regulation of cell activation	GO:0050865	0.01	LDLR, ANXA1, CD83, GPR183
COMMON UP-regulated	cellular nitrogen compound metabolic process	GO:0034641	0	ZC3H6, SARS, P2RX7, ATF5, DDIT4, KDM7A, ASNS, SLC7A11, IL31RA, CTH, SESN2, PSAT1, VEGFA, RRP7BP, SKIL, TRIB3, HLCS, ADAT2, DDIT3
	organic cyclic compound metabolic process	GO:1901360	0	ZC3H6, SARS, P2RX7, ATF5, DDIT4, KDM7A, SLC7A11, IL31RA, CTH, SESN2, PSAT1, VEGFA, RRP7BP, SKIL, TRIB3, HLCS, ADAT2, DDIT3
	negative regulation of biological process	GO:0048519	0	ZC3H6, SARS, RASA4, P2RX7, ATF5, DDIT4, ASNS, SLC7A11, IL31RA, CTH, SESN2, VEGFA, BBS2, SKIL, TRIB3, MIR628, DDIT3
	cellular nitrogen compound biosynthetic process	GO:0044271	0	ZC3H6, SARS, P2RX7, ATF5, DDIT4, KDM7A, SLC7A11, IL31RA, CTH, SESN2, PSAT1, VEGFA, SKIL, TRIB3, DDIT3
	aromatic compound biosynthetic process	GO:0019438	0	ZC3H6, SARS, P2RX7, ATF5, DDIT4, KDM7A, SLC7A11, IL31RA, CTH, SESN2, PSAT1, VEGFA, SKIL, TRIB3, DDIT3
	organic cyclic compound biosynthetic process	GO:1901362	0	ZC3H6, SARS, P2RX7, ATF5, DDIT4, KDM7A, SLC7A11, IL31RA, CTH, SESN2, PSAT1, VEGFA, SKIL, TRIB3, DDIT3
	response to chemical	GO:0042221	0	RASA4, P2RX7, DDIT4, ASNS, SLC7A11, IL31RA, PCK2, CTH, SESN2, VEGFA, BBS2, SKIL, TRIB3, HLCS, DDIT3
	negative regulation of cellular process	GO:0048523	0	ZC3H6, SARS, RASA4, P2RX7, ATF5, DDIT4, ASNS, SLC7A11, IL31RA, CTH, SESN2, VEGFA, SKIL, TRIB3, DDIT3
	system development	GO:0048731	0	SARS, P2RX7, ATF5, DDIT4, KDM7A, ASNS, SLC7A11, SPG11, IL31RA, PCK2, CTH, VEGFA, BBS2, SKIL, DDIT3
	multicellular organism development	GO:0007275	0.01	SARS, P2RX7, ATF5, DDIT4, KDM7A, ASNS, SLC7A11, SPG11, IL31RA, PCK2, CTH, VEGFA, BBS2, SKIL, DDIT3
	cellular response to chemical stimulus	GO:0070887	0	RASA4, P2RX7, DDIT4, ASNS, SLC7A11, IL31RA, PCK2, CTH, SESN2, VEGFA, BBS2, SKIL, TRIB3, DDIT3
	response to organic substance	GO:0010033	0	P2RX7, DDIT4, ASNS, SLC7A11, IL31RA, PCK2, CTH, SESN2, VEGFA, BBS2, SKIL, TRIB3, HLCS, DDIT3
	regulation of cellular biosynthetic process	GO:0031326	0	ZC3H6, SARS, P2RX7, ATF5, DDIT4, KDM7A, SLC7A11, IL31RA, CTH, SESN2, VEGFA, SKIL, TRIB3, DDIT3
	heterocycle biosynthetic process	GO:0018130	0	ZC3H6, SARS, P2RX7, ATF5, DDIT4, KDM7A, IL31RA, CTH, SESN2, PSAT1, VEGFA, SKIL, TRIB3, DDIT3
	regulation of biosynthetic process	GO:0009889	0	ZC3H6, SARS, P2RX7, ATF5, DDIT4, KDM7A, SLC7A11, IL31RA, CTH, SESN2, VEGFA, SKIL, TRIB3, DDIT3
	cellular developmental process	GO:0048869	0	P2RX7, ATF5, DDIT4, SLC7A11, SPG11, IL31RA, PCK2, CTH, VEGFA, NHSL1, BBS2, SKIL, TRIB3, DDIT3
	regulation of response to stimulus	GO:0048583	0.01	RASA4, P2RX7, DDIT4, SLC7A11, IL31RA, CTH, ULBP1, SESN2, VEGFA, BBS2, SKIL, TRIB3, DDIT3
	cellular response to organic substance	GO:0071310	0	P2RX7, DDIT4, ASNS, IL31RA, PCK2, CTH, SESN2, VEGFA, BBS2, SKIL, TRIB3, DDIT3
	negative regulation of macromolecule metabolic process	GO:0010605	0	ZC3H6, SARS, P2RX7, ATF5, DDIT4, SESN2, VEGFA, BBS2, SKIL, TRIB3, MIR628, DDIT3
	**organelle organization**	**GO:0006996**	**0.01**	**TUBE1, P2RX7, ATF5, KDM7A, SPG11, TAP2, SESN2, CUL9, VEGFA, BBS2, RRP7BP, HLCS**
	carboxylic acid metabolic process	GO:0019752	0	SARS, P2RX7, DDIT4, ASNS, SLC7A11, PCK2, CTH, SESN2, PSAT1, TRIB3, HLCS
	oxoacid metabolic process	GO:0043436	0	SARS, P2RX7, DDIT4, ASNS, SLC7A11, PCK2, CTH, SESN2, PSAT1, TRIB3, HLCS
	organic acid metabolic process	GO:0006082	0	SARS, P2RX7, DDIT4, ASNS, SLC7A11, PCK2, CTH, SESN2, PSAT1, TRIB3, HLCS
	**apoptotic process**	**GO:0006915**	**0**	**P2RX7, ATF5, DDIT4, ASNS, SLC7A11, IL31RA, CTH, VEGFA, SKIL, TRIB3, DDIT3**
	small molecule metabolic process	GO:0044281	0	SARS, P2RX7, DDIT4, ASNS, SLC7A11, PCK2, CTH, SESN2, PSAT1, TRIB3, HLCS
	programmed cell death	GO:0012501	0	P2RX7, ATF5, DDIT4, ASNS, SLC7A11, IL31RA, CTH, VEGFA, SKIL, TRIB3, DDIT3
	cell death	GO:0008219	0	P2RX7, ATF5, DDIT4, ASNS, SLC7A11, IL31RA, CTH, VEGFA, SKIL, TRIB3, DDIT3
	negative regulation of response to stimulus	GO:0048585	0	RASA4, P2RX7, DDIT4, SLC7A11, CTH, SESN2, BBS2, SKIL, TRIB3, DDIT3
	regulation of cell death	GO:0010941	0	P2RX7, ATF5, DDIT4, ASNS, SLC7A11, IL31RA, CTH, VEGFA, SKIL, DDIT3
	regulation of intracellular signal transduction	GO:1902531	0	RASA4, P2RX7, DDIT4, IL31RA, CTH, SESN2, VEGFA, SKIL, TRIB3, DDIT3
	negative regulation of gene expression	GO:0010629	0	ZC3H6, SARS, ATF5, SESN2, VEGFA, BBS2, SKIL, TRIB3, MIR628, DDIT3
	negative regulation of nitrogen compound metabolic proce	GO:0051172	0	ZC3H6, SARS, P2RX7, ATF5, DDIT4, SESN2, VEGFA, SKIL, TRIB3, DDIT3
	response to external stimulus	GO:0009605	0	P2RX7, DDIT4, ASNS, IL31RA, PCK2, SESN2, VEGFA, BBS2, HLCS, DDIT3
	negative regulation of cellular metabolic process	GO:0031324	0	ZC3H6, SARS, P2RX7, ATF5, DDIT4, SESN2, VEGFA, SKIL, TRIB3, DDIT3
	intracellular signal transduction	GO:0035556	0.01	RASA4, P2RX7, DDIT4, IL31RA, CTH, SESN2, VEGFA, SKIL, TRIB3, DDIT3
	regulation of apoptotic process	GO:0042981	0	P2RX7, ATF5, ASNS, SLC7A11, IL31RA, CTH, VEGFA, SKIL, DDIT3
	regulation of programmed cell death	GO:0043067	0	P2RX7, ATF5, ASNS, SLC7A11, IL31RA, CTH, VEGFA, SKIL, DDIT3
	negative regulation of cellular biosynthetic process	GO:0031327	0	ZC3H6, SARS, ATF5, DDIT4, SESN2, VEGFA, SKIL, TRIB3, DDIT3
	negative regulation of biosynthetic process	GO:0009890	0	ZC3H6, SARS, ATF5, DDIT4, SESN2, VEGFA, SKIL, TRIB3, DDIT3
	homeostatic process	GO:0042592	0	P2RX7, SLC7A11, IL31RA, PCK2, SESN2, VEGFA, BBS2, SKIL, DDIT3
	**cellular response to stress**	**GO:0033554**	**0**	**DDIT4, ASNS, SLC7A11, CTH, SESN2, VEGFA, SKIL, TRIB3, DDIT3**
	small molecule biosynthetic process	GO:0044283	0	P2RX7, DDIT4, ASNS, PCK2, CTH, SESN2, PSAT1, TRIB3
	negative regulation of signal transduction	GO:0009968	0	RASA4, P2RX7, DDIT4, CTH, SESN2, SKIL, TRIB3, DDIT3
	negative regulation of cell communication	GO:0010648	0	RASA4, P2RX7, DDIT4, CTH, SESN2, SKIL, TRIB3, DDIT3
	negative regulation of signaling	GO:0023057	0	RASA4, P2RX7, DDIT4, CTH, SESN2, SKIL, TRIB3, DDIT3
	cellular response to endogenous stimulus	GO:0071495	0	P2RX7, DDIT4, ASNS, PCK2, SESN2, BBS2, SKIL, TRIB3
	negative regulation of cellular macromolecule biosynthetic	GO:2000113	0	ZC3H6, SARS, ATF5, SESN2, VEGFA, SKIL, TRIB3, DDIT3
	negative regulation of nucleobase-containing compound m	GO:0045934	0	ZC3H6, SARS, ATF5, DDIT4, VEGFA, SKIL, TRIB3, DDIT3
	negative regulation of macromolecule biosynthetic process	GO:0010558	0	ZC3H6, SARS, ATF5, SESN2, VEGFA, SKIL, TRIB3, DDIT3
	response to endogenous stimulus	GO:0009719	0	P2RX7, DDIT4, ASNS, PCK2, SESN2, BBS2, SKIL, TRIB3
	organonitrogen compound biosynthetic process	GO:1901566	0.01	SARS, P2RX7, DDIT4, ASNS, SLC7A11, CTH, SESN2, PSAT1
	response to nutrient levels	GO:0031667	0	P2RX7, ASNS, PCK2, SESN2, BBS2, HLCS, DDIT3
	response to extracellular stimulus	GO:0009991	0	P2RX7, ASNS, PCK2, SESN2, BBS2, HLCS, DDIT3
	monocarboxylic acid metabolic process	GO:0032787	0	P2RX7, DDIT4, ASNS, PCK2, SESN2, TRIB3, HLCS
	**drug metabolic process**	**GO:0017144**	**0**	**P2RX7, DDIT4, ASNS, PCK2, CTH, PSAT1, HLCS**
	negative regulation of transcription, DNA-templated	GO:0045892	0	ZC3H6, SARS, ATF5, VEGFA, SKIL, TRIB3, DDIT3
	negative regulation of nucleic acid-templated transcription	GO:1903507	0	ZC3H6, SARS, ATF5, VEGFA, SKIL, TRIB3, DDIT3
	negative regulation of RNA biosynthetic process	GO:1902679	0	ZC3H6, SARS, ATF5, VEGFA, SKIL, TRIB3, DDIT3
	negative regulation of RNA metabolic process	GO:0051253	0	ZC3H6, SARS, ATF5, VEGFA, SKIL, TRIB3, DDIT3
	response to oxygen-containing compound	GO:1901700	0.01	P2RX7, DDIT4, ASNS, PCK2, SESN2, TRIB3, HLCS

Supplementary Table 5. GO-term enriched in MOLM14 and U937 metformin vs control using Genomatix

Transcriptome profiling assays on MOLM14 and U937 treated or not with 10mM metformin in independent triplicates (1 experiment). GEO: GSE97346.

Supplementary Figures

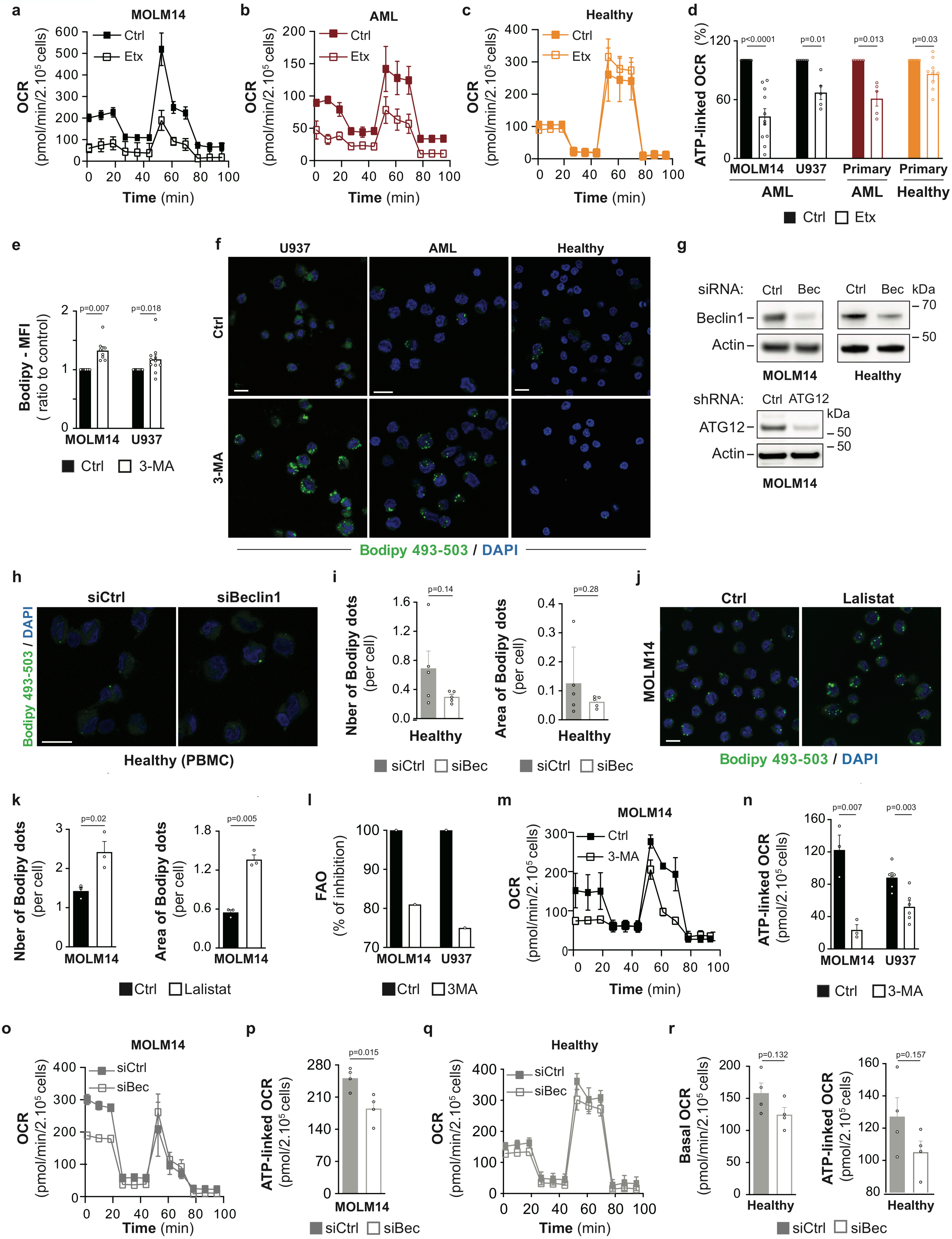
a
MOLM14
Ctrl
Etx
OCR (pmol/min/2.10⁵ cells)
Time (min)
b
AML
c
Healthy
d
ATP-linked OCR (%)
p<0.0001
p=0.01
p=0.013
p=0.03
MOLM14
U937
Primary
AML
Healthy
e
Bodipy - MFI (ratio to control)
p=0.007
p=0.018
3-MA
f
U937
AML
Healthy
Ctrl
3-MA
Bodipy 493-503 / DAPI
g
siRNA:
Ctrl
Bec
kDa
Beclin1
Actin
70
50
MOLM14
Healthy
shRNA:
ATG12
h
siCtrl
siBeclin1
Healthy (PBMC)
i
Nber of Bodipy dots (per cell)
Area of Bodipy dots (per cell)
p=0.14
p=0.28
siCtrl
siBec
j
Lalistat
k
p=0.02
p=0.005
l
FAO (% of inhibition)
3MA
m
n
ATP-linked OCR (pmol/2.10⁵ cells)
p=0.007
p=0.003
o
p
p=0.015
q
r
Basal OCR (pmol/min/2.10⁵ cells)
p=0.132
p=0.157

Supplementary Figure 1. Autophagy participates to lipid catabolism to support OxPHOS.
a-c. Data of mitochondrial respiration in MOLM14 (**a**), AML (**b**) and normal hematopoietic cells (**c**) +/- Etomoxir (Etx). **d.** Seahorse measurement of ATP-linked OCR in MOLM14 (n=11) and U937 (n=5), AML patient (n=5) and normal hematopoietic cells (PBMC n=6; CD34+ n=3) +/- Etx (One sample t test). **e.** MOLM14 and U937 cells +/- 3-methyladenine (3-MA), stained for Bodipy 493/503, and analyzed by flow cytometry, (n=8 for MOLM14 and n=11 for U937, One sample t test). **f.** U937, AML patient, and normal hematopoietic cells +/- 3-MA were stained for Bodipy 493/503 and DAPI. Confocal pictures from at least 3 independent experiments are shown. Scale bar:10µm. **g.** Western blots, from at least 3 independent experiments, showing ATG12 and Beclin1 expression after Ctrl, ATG12 or Beclin1 siRNA transfections in MOLM14 and in normal hematopoietic cells. **h.** Confocal pictures from at least 3 independent experiments of normal hematopoietic cells (PBMC) transfected with Ctrl or Beclin1 siRNAs and stained for Bodipy 493/503 and DAPI. Scale bar:10µm **i.** Histograms show the number or the area of Bodipy dots per cell. (n=5, Paired t test). **j.** Confocal pictures from 3 independent experiments of MOLM14 treated with Lalistat2 and stained for Bodipy 493/503. **k.** Histograms show the number or the area of Bodipy dots per cell. (n=3, Unpaired t test.). **l.** MOLM14 and U937 cells +/- 3-MA were examined for their rates of fatty acid β-oxidation (FAO). Data are means of triplicates. **m.** Data of mitochondrial respiration in MOLM14 cells +/- 3-MA. **n.** Seahorse measurement of ATP-linked OCR in MOLM14 (n=3) and U937 (n=6) +/- 3-MA (Unpaired t test). **o.** Data of mitochondrial respiration in MOLM14 cells after Ctrl or Beclin1 siRNA transfections. **p.** Seahorse measurement of ATP-linked OCR in MOLM14 cells after Ctrl or Beclin1 siRNA transfections. (n=4, Unpaired t test.). **q.** Data of mitochondrial respiration in primary normal hematopoietic cells (PBMC) after Ctrl or Beclin1 siRNA transfections. **r.** Seahorse measurements of basal OCR and ATP-linked OCR in normal hematopoietic cells (PBMC) after Ctrl or Beclin1 siRNA transfections (Paired t test). Data are means ± s.e.m.

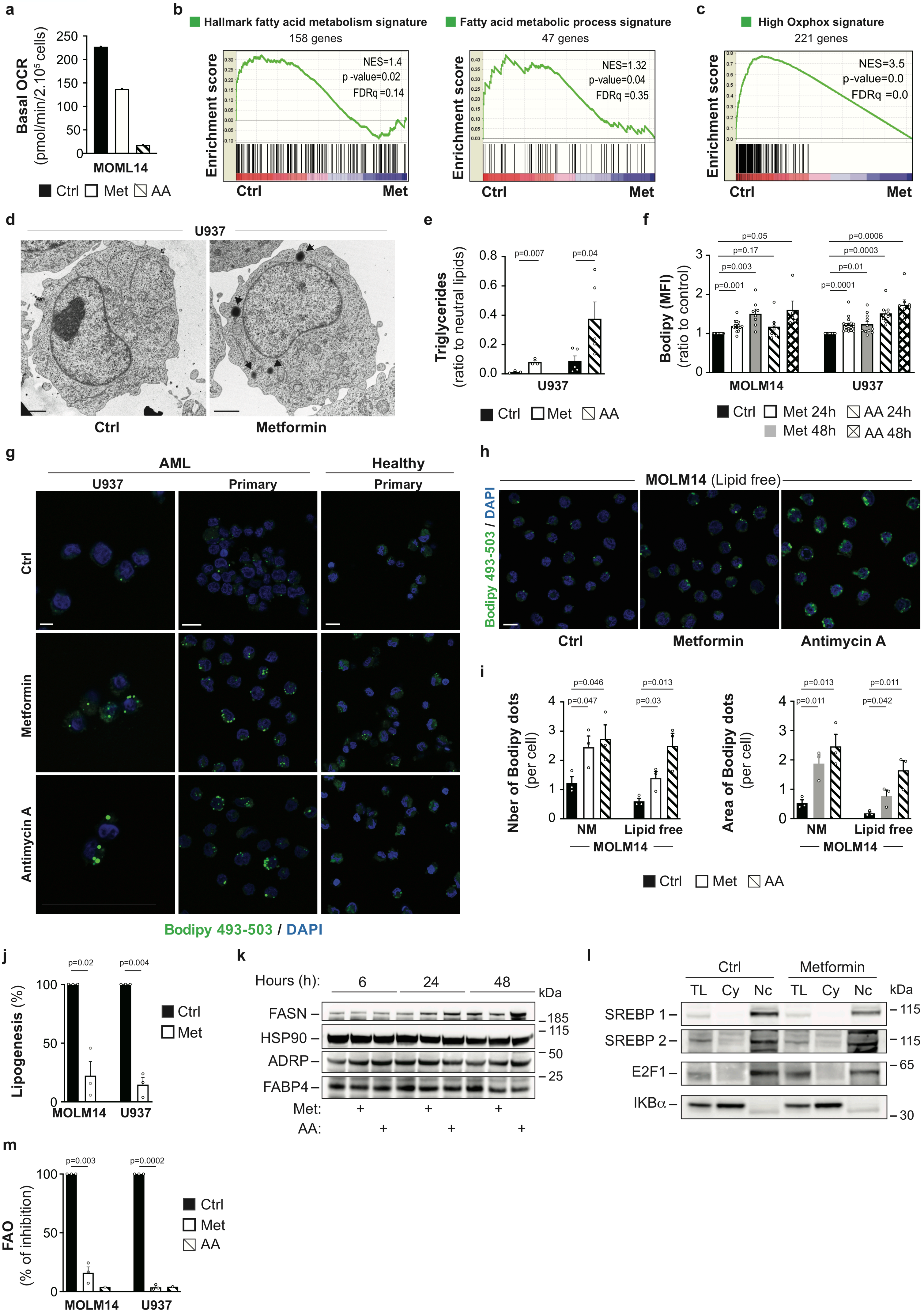
a
Basal OCR (pmol/min/2.10^5 cells)
MOML14
Ctrl Met AA
b
Hallmark fatty acid metabolism signature
158 genes
NES=1.4
p -value=0.02
FDRq =0.14
Fatty acid metabolic process signature
47 genes
NES=1.32
p-value=0.04
FDRq =0.35
Enrichment score
Ctrl
Met
c
High Oxphox signature
221 genes
NES=3.5
p-value=0.0
FDRq =0.0
d
U937
Ctrl
Metformin
e
Triglycerides (ratio to neutral lipids)
p=0.007
p=0.04
U937
Ctrl Met AA
f
Bodipy (MFI) (ratio to control)
p=0.05 p=0.17 p=0.003 p=0.001
p=0.0006 p=0.0003 p=0.01 p=0.0001
MOLM14
U937
Ctrl Met 24h AA 24h Met 48h AA 48h
g
AML
Healthy
U937
Primary
Primary
Ctrl
Metformin
Antimycin A
Bodipy 493-503 / DAPI
h
MOLM14 (Lipid free)
Bodipy 493-503 / DAPI
Ctrl
Metformin
Antimycin A
i
Nber of Bodipy dots (per cell)
p=0.046 p=0.047 p=0.013 p=0.03
Area of Bodipy dots (per cell)
p=0.013 p=0.011 p=0.011 p=0.042
NM
Lipid free
MOLM14
Ctrl Met AA
j
Lipogenesis (%)
p=0.02
p=0.004
MOLM14
U937
Ctrl
Met
k
Hours (h): 6 24 48
kDa
FASN
HSP90
ADRP
FABP4
185
115
50
25
Met:
AA:
l
Ctrl
Metformin
TL Cy Nc TL Cy Nc
kDa
SREBP 1
SREBP 2
E2F1
IKBα
115
115
65
30
m
FAO (% of inhibition)
p=0.003
p=0.0002
MOLM14
U937
Ctrl
Met
AA

Supplementary Figure 2. Inhibition of OxPHOS affects lipid metabolism.

a. Basal OCR in MOLM14 cells after metformin (Met) or antimycin A (AA) treatment for 24h. **b,c.** Gene Set Enrichment Analysis (GSEA) of fatty acid metabolism identified signatures[28] (**b**) and of High OxPHOS signature[18](**c**) were performed using transcriptomes of MOLM14 and U937 cells treated or not with metformin shown in Fig. 2a,b. (Kolmogorov-Smirnov test). **d.** U937 cells treated with metformin and processed for electron microscopy analysis. Representative electron microscopy pictures from 2 independent experiments are shown. Arrows indicate lipid droplets. Scale bar:2µm. **e.** U937 cells were treated with Met (n=3) or with AA (n=5) and processed for triglyceride content analysis (Unpaired t test). **f.** MOLM14 and U937 cells were treated or not with metformin (Met) or antimycin A (AA) for 24 and 48h, subsequently stained for Bodipy 493/503 and analysed by flow cytometry. (at least n=5 for MOLM14 and at least n=8 for U937, One sample t test). **g.** U937, primary AML patient and normal hematopoietic cells +/- met or AA, fixed and stained for Bodipy 493/503 and DAPI. Representative confocal pictures from at least 3 independent experiments are shown. Scale bar:10µm**.** **h,i.** MOLM14 cells were treated or not with met or AA in serum-free medium, fixed and stained for Bodipy 493/503 and DAPI. Representative confocal pictures from 3 independent experiments are shown. Scale:10 µm, (**h**). Histograms show the number or the area of Bodipy dots per cell. (n=3, Unpaired t test), (**i**). **j.** MOLM14 and U937 cells treated or not with Met were examined for their rates of lipogenesis. (n=3, One sample t test). **k.** Western blots showing FASN, HSP90, ADRP and FABP4 expression in MOLM14 cells treated with Met or AA for the indicated times (2 independent experiments). **l.** MOLM14 cells were treated with metformin for 48h and subjected to subcellular fractionation and western blot analysis for SREBP1, SREBP2, E2F1 and IKBα. TL:total lysate; Cy:cytosol; Nc:nucleus. (2 independent experiments). **m.** MOLM14 and U937 cells treated or not with Met (n=3) or AA (n=1) were examined for their rates of fatty acid β-oxidation (One sample t test).

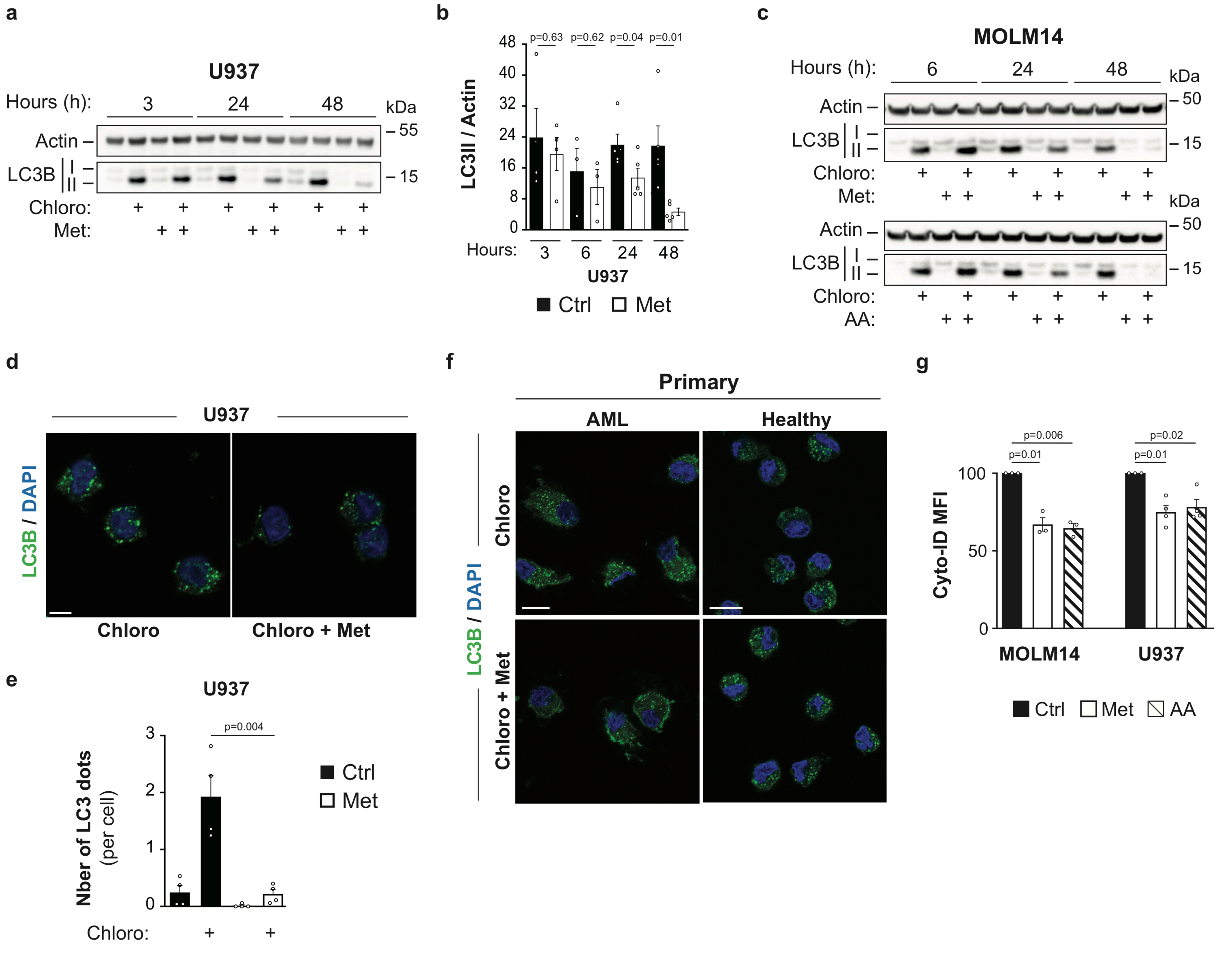

Supplementary Figure 3. Inhibition of OxPHOS reduces autophagic flux.

a. Western blots of LC3B and actin from U937 cells treated with metformin (Met) in presence or not of chloroquine (chloro) for the indicated times are shown (at least from 3 independent experiments). **b.** LC3B-II / actin ratios measured by densitometries from Western blots shown in **a.** (at least n=3, Unpaired t test.). **c.** Western blots of LC3B and actin from MOLM14 treated with metformin (Met) or antimycin A (AA) +/- chloroquine for the indicated times are shown (from at least 3 independent experiments). **d,e.** U937 cells were treated or not with metformin (Met) for 48h in presence or not of chloroquine (chloro), fixed, and stained for LC3B and DAPI. Representative confocal pictures from 4 independent experiments are shown. Scale bar:10µm (d). Histograms represent the number of LC3B puncta per cell (e), (n=4, Unpaired t test). **f.** Primary AML patient cells and normal hematopoietic cells (PBMC) were treated +/- metformin and stained for LC3B and DAPI. Representative confocal pictures from at least 5 independent experiments are shown. Scale:10µm. **g.** MOLM14 (n=3) and U937 (n=4) cells were treated with metformin or antimycin A for 48h and stained with Cyto-ID before flow cytometry analysis (One sample t test).

Data are means ± s.e.m.

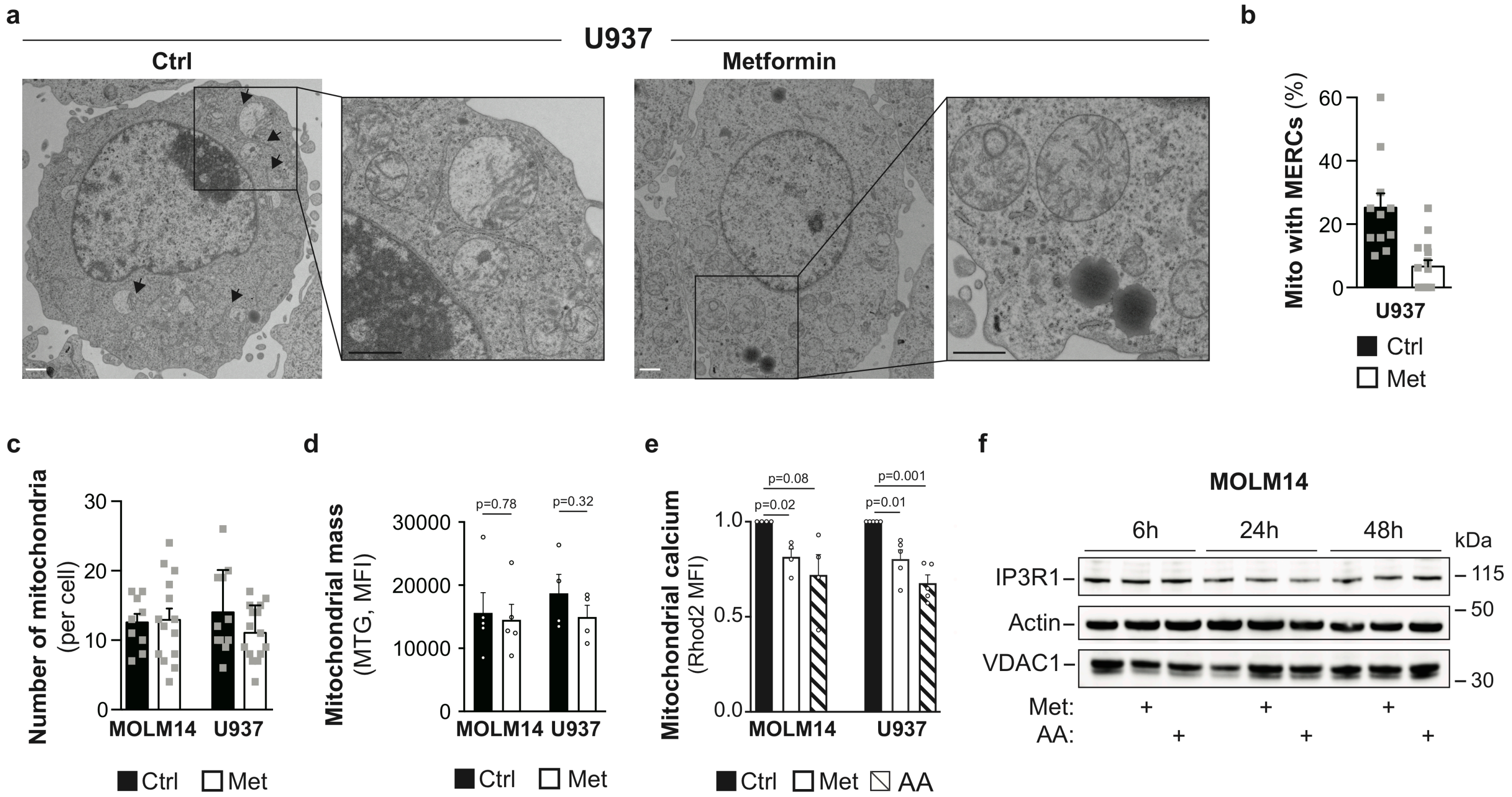

Supplementary Figure 4. OxPHOS regulates MERCs number and function.

a. U937 cells treated with metformin for 24h were processed for electron microscopy analysis. Representative electron microscopy pictures from 2 independent experiments are shown. Arrows indicate MERCs. Scale bar: 1µm. **b.** Histograms represent the % of mitochondria that are in contact with endoplasmic reticulum per cell treated or not with metformin from pictures obtained in (**a**). Data are means ± s.e.m with each dot corresponding to one cell. **c.** Histograms represent the number of mitochondria per cell treated or not with metformin from pictures obtained in (**a**). **d.** MOLM14 and U937 cells treated with metformin for 24h were stained with MitoTracker™ Green FM (MTG) to determine mitochondrial mass by flow cytometry analysis. (MOLM14 n=5; U937 n=4, Unpaired t test). **e.** MOLM14 and U937 cells treated with metformin (Met) or antimycin A (AA) for 24h were stained with Rhod2 to determine mitochondrial calcium by flow cytometry analysis (MOLM14 n=4; U937 n=5, Unpaired t test). **f.** Western blots of IP3R1, VDAC1 and actin from MOLM14 treated with metformin (Met) or antimycin A (AA) for the indicated times are shown (from 2 independent experiments).

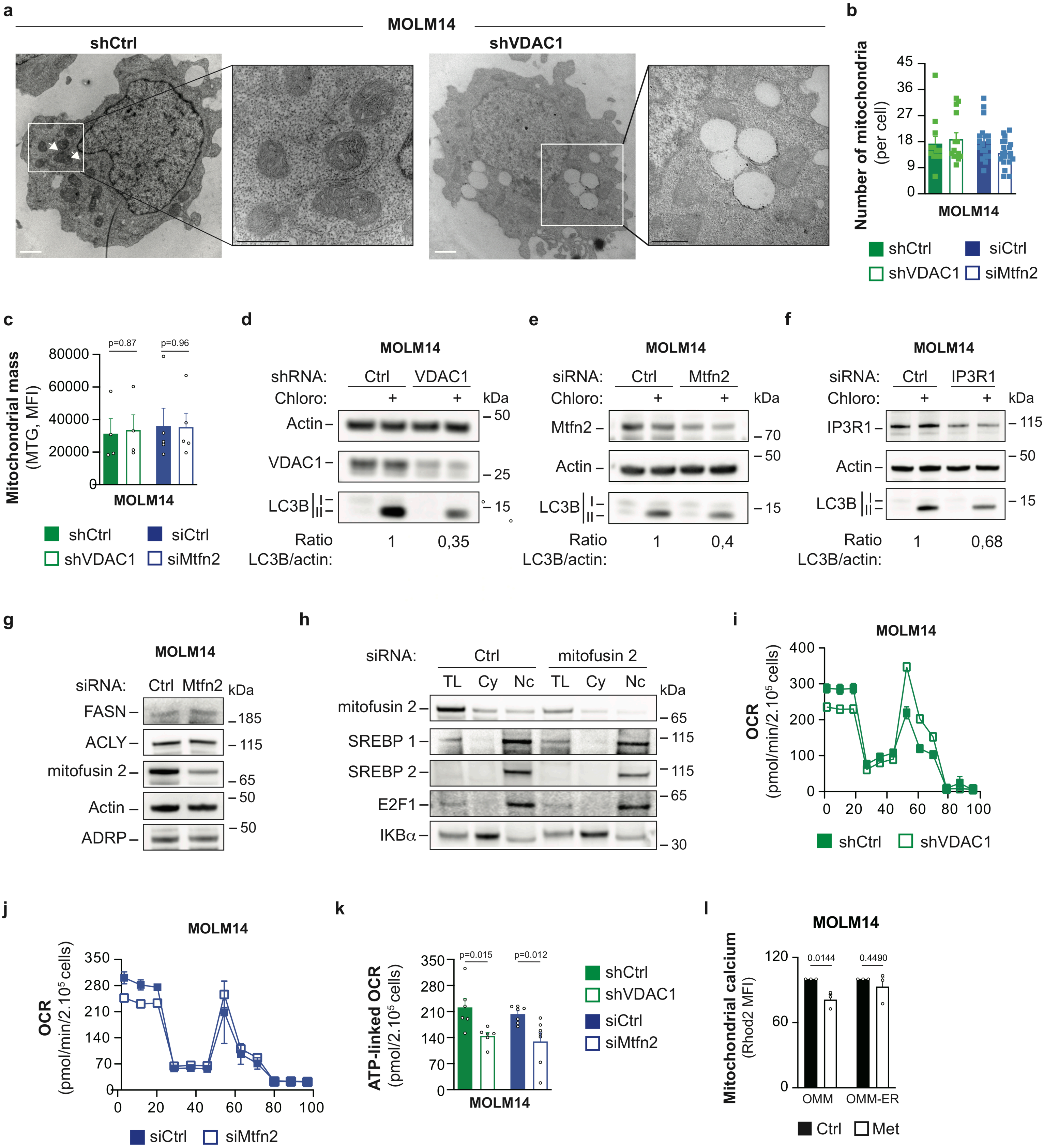
a
MOLM14
shCtrl
shVDAC1
b
Number of mitochondria (per cell)
45
36
27
18
9
0
MOLM14
shCtrl
siCtrl
shVDAC1
siMtfn2
c
Mitochondrial mass (MTG, MFI)
80000
60000
40000
20000
0
p=0.87
p=0.96
MOLM14
shCtrl
siCtrl
shVDAC1
siMtfn2
d
MOLM14
shRNA: Ctrl VDAC1
Chloro: + +
kDa
Actin
VDAC1
LC3B I II
50
25
15
Ratio LC3B/actin: 1 0,35
e
MOLM14
siRNA: Ctrl Mtfn2
Chloro: + +
kDa
Mtfn2
Actin
LC3B I II
70
50
15
Ratio LC3B/actin: 1 0,4
f
MOLM14
siRNA: Ctrl IP3R1
Chloro: + +
kDa
IP3R1
Actin
LC3B I II
115
50
15
Ratio LC3B/actin: 1 0,68
g
MOLM14
siRNA: Ctrl Mtfn2
kDa
FASN
ACLY
mitofusin 2
Actin
ADRP
185
115
65
50
50
h
siRNA: Ctrl mitofusin 2
TL Cy Nc TL Cy Nc
kDa
mitofusin 2
SREBP 1
SREBP 2
E2F1
IKBα
65
115
115
65
30
i
MOLM14
OCR (pmol/min/2.10^5 cells)
400
300
200
100
0
0 20 40 60 80 100
shCtrl
shVDAC1
j
MOLM14
OCR (pmol/min/2.10^5 cells)
350
280
210
140
70
0
0 20 40 60 80 100
siCtrl
siMtfn2
k
ATP-linked OCR (pmol/2.10^5 cells)
350
280
210
140
70
0
p=0.015
p=0.012
MOLM14
shCtrl
shVDAC1
siCtrl
siMtfn2
l
MOLM14
Mitochondrial calcium (Rhod2 MFI)
120
60
0
0.0144
0.4490
OMM
OMM-ER
Ctrl
Met

Supplementary Figure 5. MERCs regulate lipid degradation through autophagy to sustain OxPHOS.
a. MOLM14 cells transduced with Ctrl or VDAC1 shRNAs +/- metformin processed for electron microscopy analysis. Electron microscopy pictures (1 experiment) are shown. Arrows indicate MERCs. Scale bar:1µm. **b.** Histograms represent the number of mitochondria per MOLM14 cell transduced with Ctrl or VDAC1 shRNAs obtained in (**a**) or per MOLM14 cell transfected with control (Ctrl) or mitofusin 2 (Mtfn2) siRNAs obtained in **Fig. 5a**. **c.** MOLM14 cells transduced with Ctrl or VDAC1 shRNAs (n=4) or transfected with Ctrl or Mtfn2 siRNAs (n=5) were stained with MitoTracker™ Green FM (MTG) to determine mitochondrial mass by flow cytometry (Unpaired t test). **d.** Western blots showing VDAC1, LC3B and actin expression after Ctrl or VDAC1 shRNA transduction in MOLM14 cells +/- chloro. Numbers indicate the ratio of LC3B/actin (2 independent experiments). **e.** Western blots showing Mtfn2, LC3B and actin expression after Ctrl or Mtfn2 siRNA transfections in MOLM14 cells +/- chloro. Numbers indicate the ratio of LC3B/actin (2 independent experiments). **f.** Western blots showing IP3R1, LC3B and actin expression after Ctrl or IP3R1 siRNA transfections in MOLM14 cells +/- chloro. Numbers indicate the ratio of LC3B/actin (1 experiment). **g.** Western blots of the fatty acid synthase (FASN), ATP citrate synthase (ACLY), Mtfn2, adipophilin (ADRP) and actin after Ctrl or Mtfn2 siRNA transfections in MOLM14 cells (2 independent experiments). **h.** MOLM14 cells transfected with Ctrl or Mtfn2 siRNAs were treated with metformin and subjected to subcellular fractionation and western blot analysis for Mtfn2, SREBP1, SREBP2, E2F1 and IKBα. TL: total lysate; Cy: cytosol; Nc: nucleus. (2 independent experiments). **i,j.** Data of mitochondrial respiration after Ctrl or VDAC1 shRNA transduction (**i**) or Ctrl or Mtfn2 siRNA transfections (**j**) in MOLM14 cells. **k.** Measurement of ATP-linked OCR in MOLM14 cells transduced with Ctrl or VDAC1 shRNAs (n=6) or transfected with Ctrl or Mtfn2 siRNAs (n=7) (Unpaired t test). **l.** MOLM14 cells were transduced with the mitochondria-ER organelle linker (OMM-ER) or its control sequence (OMM), treated with metformin and stained with Rhod2 to determine mitochondrial calcium by flow cytometry. (n=3, One sample t test).
Data are means ± s.e.m.

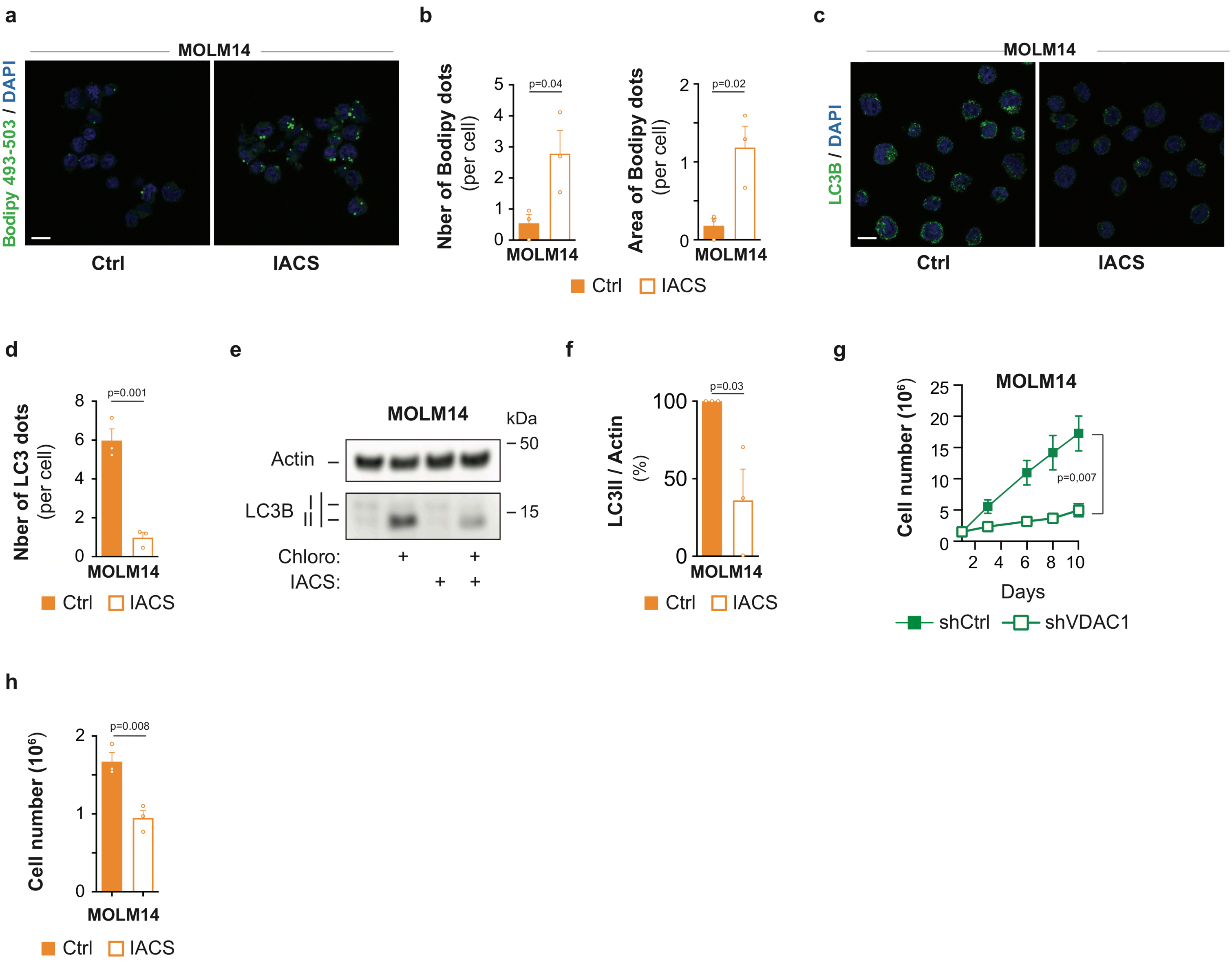

Supplementary Figure 6. MERCs support the dialogue between autophagy and OxPHOS.

a.b. MOLM14 cells were treated or not with IACS-010759 (100nM) for 48h, fixed and stained for Bodipy 493/503 and DAPI. Representative confocal pictures from 3 independent experiments are shown. Scale bar: 10µm (a). Histograms show the number or the area of Bodipy 493/503 dots per cell (b). (n=3, Unpaired t test). **c,d.** MOLM14 cells were treated or not with IACS-010759 (100nM) for 48h, fixed and stained for LC3B and DAPI. Representative confocal pictures from 3 independent experiments are shown. Scale bar: 10µm (c). Histograms show the number of dots per cell (d). (n=3, Unpaired t test). **e,f.** Western blots showing LC3B and actin expression of MOLM14 cells treated or not with IACS-010759 (100nM) for 48h (e) (3 independent experiments). Quantification of western blots (f). (n=3, Unpaired t test). **g.** MOLM14 cells were transduced with Ctrl or VDAC1 shRNAs, and the number of cells was assessed by trypan blue exclusion counting. The graph represents the cumulative number of cells per day. (n=5, Mann Whitney test). **h.** MOLM14 cells were treated or not with IACS-010759 (100nM) for 48h, and the number of cells was assessed by trypan blue exclusion counting (n=3, Unpaired t test).

Data are means ± s.e.m.

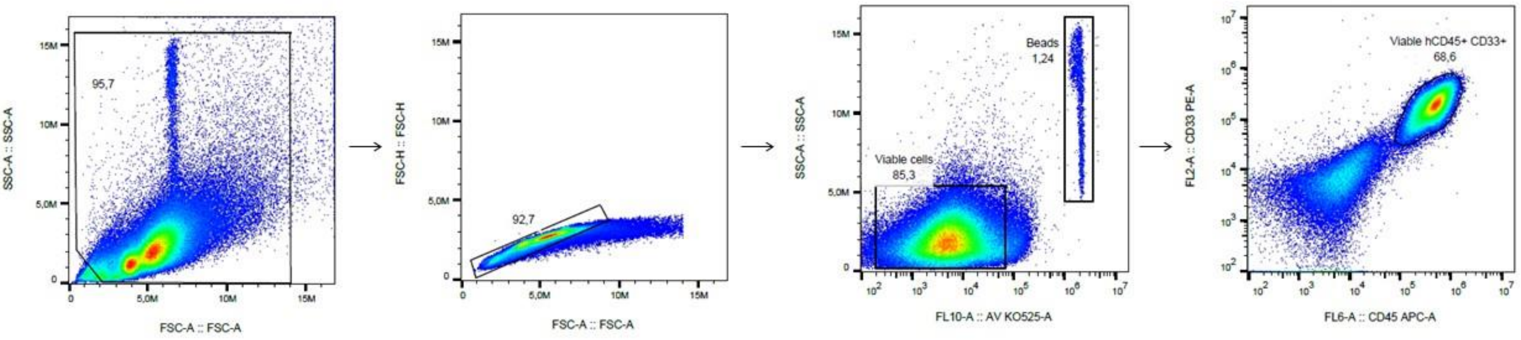

Supplementary Figure 7. Gating strategy for flow cytometry analysis.

Cells from the bone marrow compartment of NSG mice are selected according to the forward scatter (FSC) and side scatter (SSC) area (A) parameters. Then, doublet exclusion is performed using height (H) versus area (A) parameters of FSC and live/dead discrimination is applied using annexinV dye. The AML blast population is CD45+CD33+. The tumour burden is determined using the beads and blasts true count.

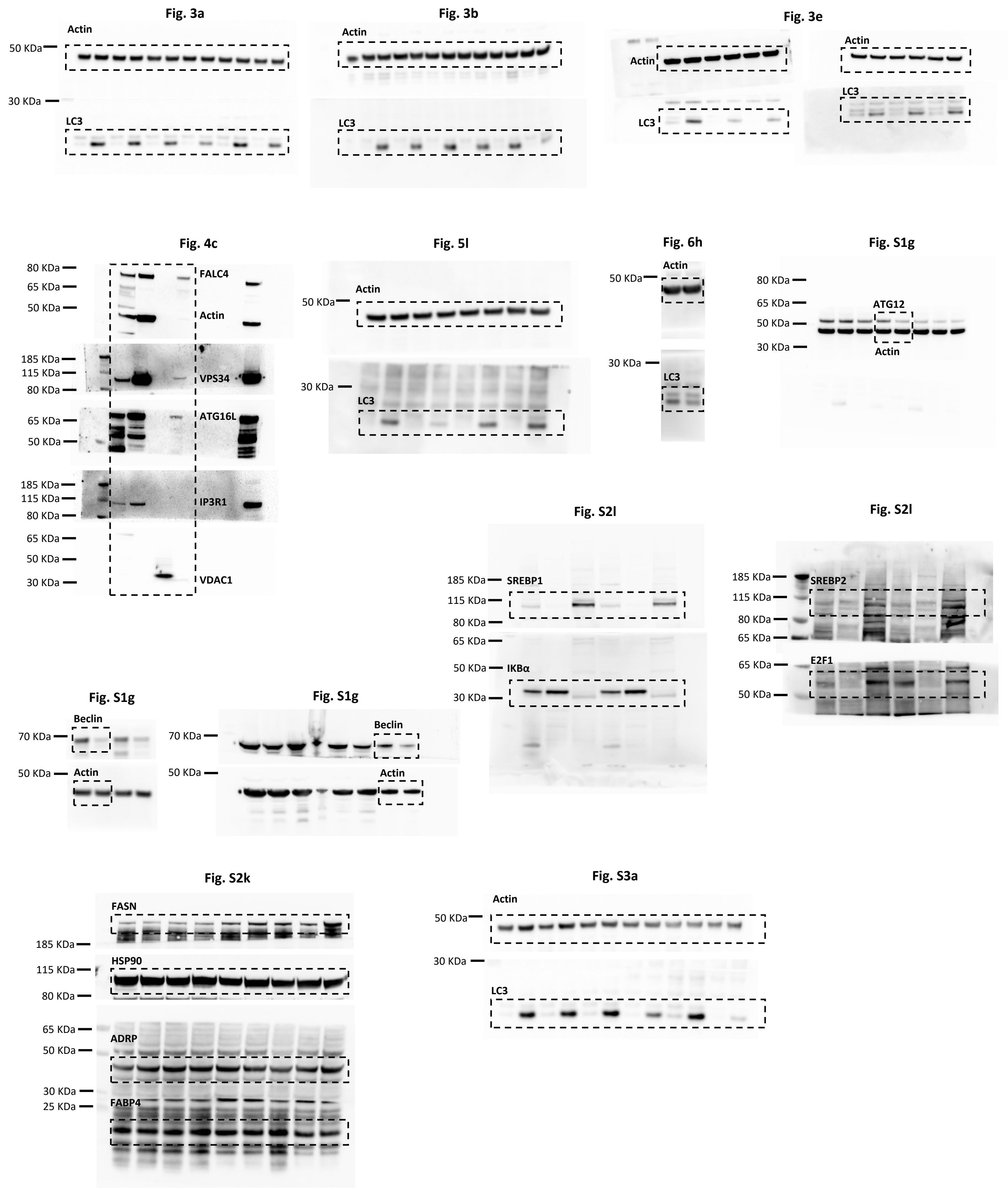

Supplementary Figure 8. Full scans of uncropped blots

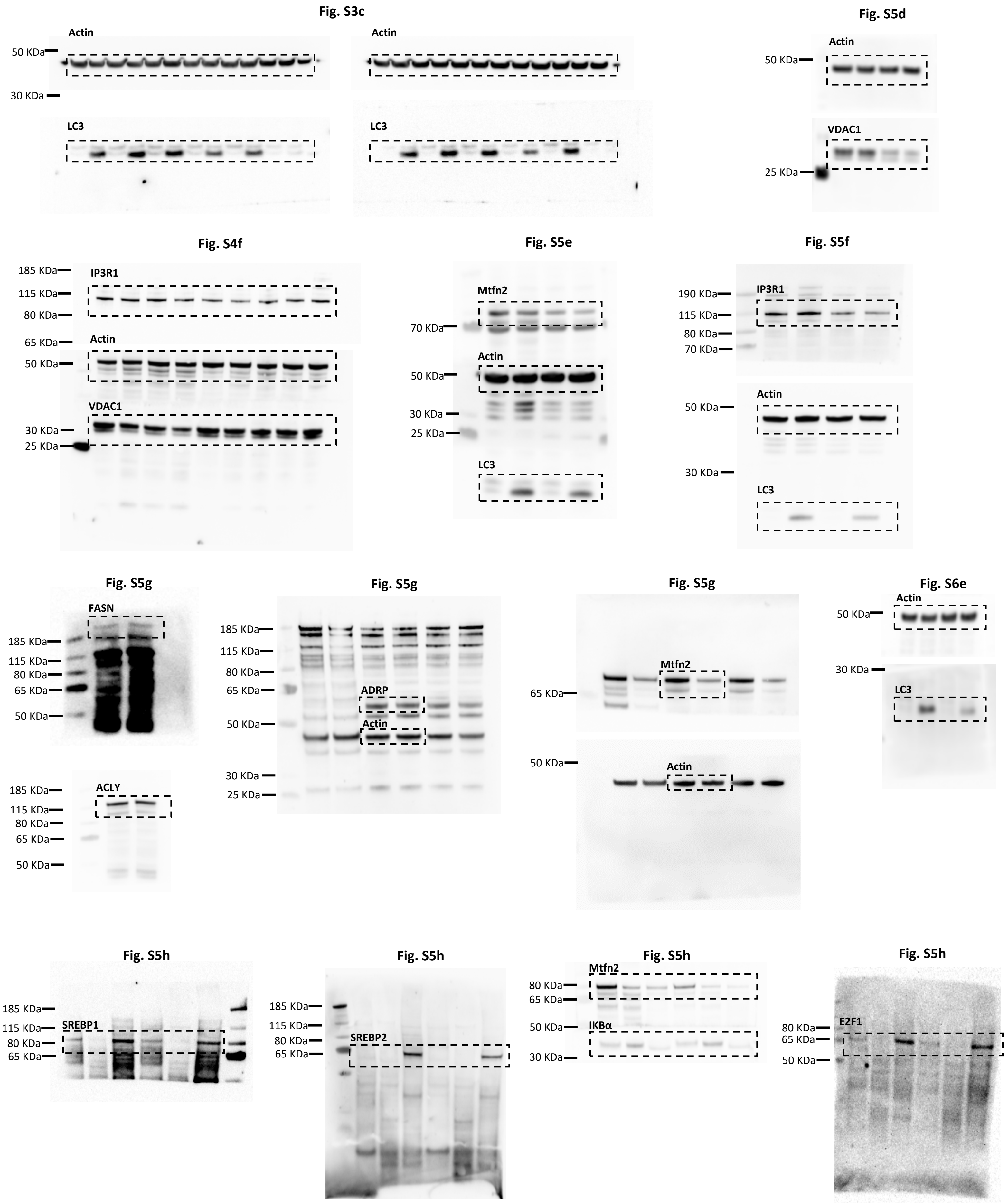

Supplementary Figure 8. Continued Full scans of uncropped blots
