# Supplementary material for: Autophagy regulates fatty acid availability for oxidative phosphorylation through mitochondria-endoplasmic reticulum contact sites
Source: Nat Commun. 2020 Aug 13;11:4056. doi: 10.1038/s41467-020-17882-2 (PMC7426880; doi:10.1038/s41467-020-17882-2)
Supplement: Supplementary file 3 — Reporting Summary [file 41467_2020_17882_MOESM3_ESM.pdf]

nature research

Corresponding author(s): *Carine Joffre*
Jean-Emmanuel Sarry

Last updated by author(s): *Jul 1, 2020*

Reporting Summary

Nature Research wishes to improve the reproducibility of the work that we publish. This form provides structure for consistency and transparency in reporting. For further information on Nature Research policies, see our Editorial Policies and the Editorial Policy Checklist.

Statistics

For all statistical analyses, confirm that the following items are present in the figure legend, table legend, main text, or Methods section.

n/a	Confirmed	
☐	☒	The exact sample size (n) for each experimental group/condition, given as a discrete number and unit of measurement
☐	☒	A statement on whether measurements were taken from distinct samples or whether the same sample was measured repeatedly
☐	☒	The statistical test(s) used AND whether they are one- or two-sided<br>*Only common tests should be described solely by name; describe more complex techniques in the Methods section.*
☒	☐	A description of all covariates tested
☒	☐	A description of any assumptions or corrections, such as tests of normality and adjustment for multiple comparisons
☐	☒	A full description of the statistical parameters including central tendency (e.g. means) or other basic estimates (e.g. regression coefficient) AND variation (e.g. standard deviation) or associated estimates of uncertainty (e.g. confidence intervals)
☐	☒	For null hypothesis testing, the test statistic (e.g. F, t, r) with confidence intervals, effect sizes, degrees of freedom and P value noted<br>*Give P values as exact values whenever suitable.*
☒	☐	For Bayesian analysis, information on the choice of priors and Markov chain Monte Carlo settings
☒	☐	For hierarchical and complex designs, identification of the appropriate level for tests and full reporting of outcomes
☒	☐	Estimates of effect sizes (e.g. Cohen's d, Pearson's r), indicating how they were calculated

Our web collection on statistics for biologists contains articles on many of the points above.

Software and code

Policy information about availability of computer code

Data collection	*GSEA 3.0 Software (http://software.broadinstitute.org/gsea/index.jsp)*<br>*GeneSys Software (https://www.syngene.com/support/software-downloads/)*<br>*Zen Black2012 SP2 (https://www.zeiss.fr/microscopie/produits/microscope-software/zen-lite/zen-2-lite-download.html)*
Data analysis	*Prism 6.0 Software (https://www.graphpad.com/scientific-software/prism/)*<br>*ImageJ 2.0 Software (https://imagej.nih.gov/ij/download.html)*<br>*GeneTool Software (https://www.syngene.com/support/software-downloads/)*<br>*Genomatix Software (https://www.genomatix.de/)*<br>*FlowJo 10.4.2 Software (https://www.flowjo.com/solutions/flowjo/downloads)*<br>*Wave Desktop 2.6 (https://www.agilent.com/en/products/cell-analysis/software-download-for-wave-desktop)*

For manuscripts utilizing custom algorithms or software that are central to the research but not yet described in published literature, software must be made available to editors and reviewers. We strongly encourage code deposition in a community repository (e.g. GitHub). See the Nature Research guidelines for submitting code & software for further information.

Data

Policy information about availability of data

All manuscripts must include a data availability statement. This statement should provide the following information, where applicable:
- Accession codes, unique identifiers, or web links for publicly available datasets
- A list of figures that have associated raw data
- A description of any restrictions on data availability

Transcriptome profiling assays on MOLM-14 and U937 treated or not with 10mM metformin in independent triplicates. GEO: GSE97346 (https://

www.ncbi.nlm.nih.gov/geo/query/acc.cgi?acc=GSE97346).
Raw data is available on reasonable request. The source data for Fig. 1–6 and Supplementary Fig. 1-6 are provided in the Source Data file.

Field-specific reporting

Please select the one below that is the best fit for your research. If you are not sure, read the appropriate sections before making your selection.

- [x] Life sciences
- [ ] Behavioural & social sciences
- [ ] Ecological, evolutionary & environmental sciences

For a reference copy of the document with all sections, see nature.com/documents/nr-reporting-summary-flat.pdf

Life sciences study design

All studies must disclose on these points even when the disclosure is negative.

Sample size	No statistical methods were used to predetermine sample size. Independent experiments were performed at least third time to allow statistical analysis. N=3-6 for cell line analysis and 3-10 for primary cell analysis based on the availability of primary samples.
Data exclusions	No data obtained from experiments performed on cell lines were excluded.<br>Only one data obtained from one patient was excluded since basal value (in control conditions) was 5 times higher than the value of the standard error.
Replication	Experiments were replicated thrice with technical replicates for the OCR analysis and and at least three times with biological replicates using identical or similar conditions and reagents to ensure the results were reproducible and representative.<br>All experiments obtained comparable results, replicates were successful.
Randomization	Mice xenografted with either MOLM14 cells expressing shRNA control or shRNA VDAC1 were randomly distributed between arms taking into account the weight of mice and their gender. Treatment groups (IACS-010759 vs Placebo) were randomly assigned upon the weight of mice and their gender.
Blinding	The investigator responsible for in vivo experiments was blinded for the group allocation and at point of analysis, but blinding was not possible during IACS-010759 since this compound is opaque and white while the vehicle is transparent.<br>For expression analysis, blinding was not possible to enable orderly loading of the gels. For immunofluorescence analysis staining investigators were blinded.<br>Other experiments presented in this study not required blinding.

Behavioural & social sciences study design

All studies must disclose on these points even when the disclosure is negative.

Study description	Briefly describe the study type including whether data are quantitative, qualitative, or mixed-methods (e.g. qualitative cross-sectional, quantitative experimental, mixed-methods case study).
Research sample	State the research sample (e.g. Harvard university undergraduates, villagers in rural India) and provide relevant demographic information (e.g. age, sex) and indicate whether the sample is representative. Provide a rationale for the study sample chosen. For studies involving existing datasets, please describe the dataset and source.
Sampling strategy	Describe the sampling procedure (e.g. random, snowball, stratified, convenience). Describe the statistical methods that were used to predetermine sample size OR if no sample-size calculation was performed, describe how sample sizes were chosen and provide a rationale for why these sample sizes are sufficient. For qualitative data, please indicate whether data saturation was considered, and what criteria were used to decide that no further sampling was needed.
Data collection	Provide details about the data collection procedure, including the instruments or devices used to record the data (e.g. pen and paper, computer, eye tracker, video or audio equipment) whether anyone was present besides the participant(s) and the researcher, and whether the researcher was blind to experimental condition and/or the study hypothesis during data collection.
Timing	Indicate the start and stop dates of data collection. If there is a gap between collection periods, state the dates for each sample cohort.
Data exclusions	If no data were excluded from the analyses, state so OR if data were excluded, provide the exact number of exclusions and the rationale behind them, indicating whether exclusion criteria were pre-established.
Non-participation	State how many participants dropped out/declined participation and the reason(s) given OR provide response rate OR state that no participants dropped out/declined participation.
Randomization	If participants were not allocated into experimental groups, state so OR describe how participants were allocated to groups, and if allocation was not random, describe how covariates were controlled.

Ecological, evolutionary & environmental sciences study design

All studies must disclose on these points even when the disclosure is negative.

Study description	*Briefly describe the study. For quantitative data include treatment factors and interactions, design structure (e.g. factorial, nested, hierarchical), nature and number of experimental units and replicates.*
Research sample	*Describe the research sample (e.g. a group of tagged Passer domesticus, all Stenocereus thurberi within Organ Pipe Cactus National Monument), and provide a rationale for the sample choice. When relevant, describe the organism taxa, source, sex, age range and any manipulations. State what population the sample is meant to represent when applicable. For studies involving existing datasets, describe the data and its source.*
Sampling strategy	*Note the sampling procedure. Describe the statistical methods that were used to predetermine sample size OR if no sample-size calculation was performed, describe how sample sizes were chosen and provide a rationale for why these sample sizes are sufficient.*
Data collection	*Describe the data collection procedure, including who recorded the data and how.*
Timing and spatial scale	*Indicate the start and stop dates of data collection, noting the frequency and periodicity of sampling and providing a rationale for these choices. If there is a gap between collection periods, state the dates for each sample cohort. Specify the spatial scale from which the data are taken*
Data exclusions	*If no data were excluded from the analyses, state so OR if data were excluded, describe the exclusions and the rationale behind them, indicating whether exclusion criteria were pre-established.*
Reproducibility	*Describe the measures taken to verify the reproducibility of experimental findings. For each experiment, note whether any attempts to repeat the experiment failed OR state that all attempts to repeat the experiment were successful.*
Randomization	*Describe how samples/organisms/participants were allocated into groups. If allocation was not random, describe how covariates were controlled. If this is not relevant to your study, explain why.*
Blinding	*Describe the extent of blinding used during data acquisition and analysis. If blinding was not possible, describe why OR explain why blinding was not relevant to your study.*

Did the study involve field work? ☐ Yes ☐ No

Field work, collection and transport

Field conditions	*Describe the study conditions for field work, providing relevant parameters (e.g. temperature, rainfall).*
Location	*State the location of the sampling or experiment, providing relevant parameters (e.g. latitude and longitude, elevation, water depth).*
Access & import/export	*Describe the efforts you have made to access habitats and to collect and import/export your samples in a responsible manner and in compliance with local, national and international laws, noting any permits that were obtained (give the name of the issuing authority, the date of issue, and any identifying information).*
Disturbance	*Describe any disturbance caused by the study and how it was minimized.*

Reporting for specific materials, systems and methods

We require information from authors about some types of materials, experimental systems and methods used in many studies. Here, indicate whether each material, system or method listed is relevant to your study. If you are not sure if a list item applies to your research, read the appropriate section before selecting a response.

Materials & experimental systems

n/a	Involved in the study	
☐	☒	Antibodies
☐	☒	Eukaryotic cell lines
☒	☐	Palaeontology and archaeology
☐	☒	Animals and other organisms
☐	☒	Human research participants
☒	☐	Clinical data
☒	☐	Dual use research of concern

Methods

n/a	Involved in the study	
☒	☐	ChIP-seq
☐	☒	Flow cytometry
☒	☐	MRI-based neuroimaging

Antibodies

Antibodies used	*Rabbit antibodies against LC3B (#2775, Cell signalling and #PM036, MBL), ATG12 (#4180, Cell signaling), FASN (#3180, Cell signaling), Beclin1 (#3738 Cell signaling), E2F1 (KH95, Santa Cruz Biotechnology), ACLY (HPA022434, Atlas Antibodies), SREBP2 (PA1-338, Invitrogen), TOMM20 (GTX133756, GeneTex).*<br><br>*Mouse antibodies against HSP90 (#4874, Cell signaling), IP3R1 (sc-271197, Santa Cruz Biotechnology), actin (MAB1501, Millipore), VDAC1 (ab15865, Millipore), ADRP (610102, PROGEN), SREPB1 (NB600-582, Novus Biologicals), hCD45 (Clone 2D1, 641417).*<br><br>*Goat antibody against FABP4 (AF3150, R&D System)*
Validation	*LC3B (2775): validation for WB by manufacturer (transfection with exogenous LC3B and +/- treatment with chloroquine). Reactivity for Human, Mousse and Rat.*<br><br>*LC3B (#PM036, MBL): validation for WB by manufacturer (In normal MEF cells and ATG5-/-). Reactivity for Human, Mousse, Rat and Hamster.*<br><br>*ATG12 (4180): validation for WB by manufacturer (expression in different cell lines) and specificity provided in the manuscript by using shRNA. Reactivity for Human, Mousse, Rat and Monkey.*<br><br>*Beclin1 (3738): validation for WB by manufacturer (expression in different cell lines) and specificity provided in the manuscript by using siRNA. Reactivity for Human, Mousse and Rat.*<br><br>*IP3R1 (sc-271197): validation for WB and IF by manufacturer and in literature for PLA application (DOI: 10.1016/j.immuni.2018.02.012). Reactivity for Human, Mousse and Rat.*<br><br>*Actin (MAB1501): validation for WB by manufacturer. Reactivity for Human, Mousse and Rat.*<br><br>*VDAC1 (ab15865): validation for WB and IF by manufacturer and in the manuscript for PLA and WB application by using shRNA against VDAC1. Reactivity for Human, Mousse and Rat.*<br><br>*hCD45 (Clone 2D1, 641417): validation for flow cytometry by manufacturer. Reactivity for Human.*<br><br>*FASN (#3180, Cell signaling): validation for WB and IF by manufacturer (expression in different cell lines). Reactivity for Human, Mousse and Rat.*<br><br>*E2F1 (KH95, Santa Cruz Biotechnology): validation for WB and IF by manufacturer (expression in different cell lines in whole cell lysate and nuclear extracts). Reactivity for Human, Mousse and Rat.*<br><br>*ACLY (HPA022434, Atlas Antibodies): validation for WB by manufacturer (expression in A-549 cells transfected with control siRNA, target specific siRNA probe). Reactivity for Human, Mousse and Rat.*<br><br>*SREBP2 (PA1-338, Invitrogen): validation for WB and IF by manufacturer (expression in different cell lines in whole cell lysate and nuclear extracts). Reactivity for Human, Mousse and Rat.*<br><br>*TOMM20 (GTX133756, GeneTex): validation for WB by manufacturer (expression in different cell lines and with the use of a competitor's antibody) and specificity for IF is provided in the manuscript by using a super resolution confocal (LSM 880 FastAiryScan) showing mitochondrial staining. Reactivity for Human, Mousse and Rat.*<br><br>*HSP90 (#4874, Cell signaling): validation for WB by manufacturer (expression in different cell lines). Reactivity for Human, Mousse, Rat, Monkey, D.melanogaster, and Zebrafish.*<br><br>*ADRP (610102, PROGEN): validation for WB by manufacturer (expression in different cell lines and primary samples). Reactivity for Human, Rat and Dog.*<br><br>*SREPB1 (NB600-582, Novus Biologicals): validation for WB by manufacturer (expression in different cell lines in cytosolic and nuclear extracts). Reactivity for Human, Rat, Dog Chicken and Hamster.*<br><br>*FABP4 (AF3150, R&D System): validation for WB by manufacturer (expression in different cell lines). Reactivity for Human.*

Eukaryotic cell lines

Policy information about cell lines

Cell line source(s)	*MOLM14 cells were purchased from DSMZ and U937 cells were purchased from ATCC.*
Authentication	*DSMZ and ATCC provide authenticated cell lines by cytochrome C oxidase I gene analysis and short tandem repeat profiling. The names of the used cell lines are authentic and previously published.*

Mycoplasma contamination	*All cell lines were regularly tested for absence of mycoplasma contamination by using the MycoAlert Mycoplasma Detection Kit (Lonza #LT07-703). All cell lines used in this study were negative for mycoplasma.*
Commonly misidentified lines (See ICLAC register)	*No commonly misidentified lines were used in this study.*

Palaeontology and Archaeology

Specimen provenance	*Provide provenance information for specimens and describe permits that were obtained for the work (including the name of the issuing authority, the date of issue, and any identifying information).*
Specimen deposition	*Indicate where the specimens have been deposited to permit free access by other researchers.*
Dating methods	*If new dates are provided, describe how they were obtained (e.g. collection, storage, sample pretreatment and measurement), where they were obtained (i.e. lab name), the calibration program and the protocol for quality assurance OR state that no new dates are provided.*

☐ Tick this box to confirm that the raw and calibrated dates are available in the paper or in Supplementary Information.

Ethics oversight	*Identify the organization(s) that approved or provided guidance on the study protocol, OR state that no ethical approval or guidance was required and explain why not.*

Note that full information on the approval of the study protocol must also be provided in the manuscript.

Animals and other organisms

Policy information about studies involving animals; ARRIVE guidelines recommended for reporting animal research

Laboratory animals	*Animals were used for transplantation of AML cell lines in accordance with a protocol reviewed and approved by the Institutional Animal Care and Use Committee of Région MidiPyrénées (France). NOD/LtSz-SCID/IL-2Rγchain null (NSG) mice were produced at the Genotoul Anexplo platform at Toulouse (France) using breeders obtained from Charles River Laboratories. Mice were housed in sterile conditions using HEPA-filtered microisolators and fed with irradiated food and sterile water. 8 weeks old male and female mice (1:1) were sublethally treated with busulfan (20 mg/kg) 24 hours before injection of AML cells.*
Wild animals	*The study did not involve wild animals.*
Field-collected samples	*The study did not involve samples collected from field.*
Ethics oversight	*This study was approved by the Institutional Animal Care and Use Committee of Région Midi- Pyrénées (France)*

Note that full information on the approval of the study protocol must also be provided in the manuscript.

Human research participants

Policy information about studies involving human research participants

Population characteristics	*Population characteristics correspond to adult AML patients (Toulouse university Hospital, France) or healthy adult donors (Etablissement Francais du Sang, Toulouse, France) collected between 2015 and 2019.*<br>*Human AML cells used in this study were histopathologically and clinically diagnosed. Among these patients, there are 10 male and 10 female patients. The age of patients ranges from 22 to 78 years old.*<br>*Non leukemia controls (PBMC and CD34+) were obtained from EFS (Etablissement Français du Sang) BFC and Occitanie. PBMC and CD34+ were purified from both gender healthy donors blood.*
Recruitment	*Primary AML patient cells have been collected during routine diagnostic procedures at the Toulouse University Hospital (TUH), after informed consent.*<br>*Non leukemia controls (PBMC and CD34+) were obtained from blood samples of healthy donor (Etablissement Français du Sang, Toulouse, France).*<br>*The selection of patients was random and we believe that there was no bias in results.*
Ethics oversight	*Primary AML patient cells have been stored at the HIMIP collection (BB-0033-00060). According to the French law, HIMIP collections has been declared to the Ministry of Higher Education and Research (DC 2008-307 collection 1) and obtained a transfer agreement (AC 2008-129) after approbation by the "Comite"de Protection des Personnes Sud-Ouest et Outremer II" (ethical committee). Clinical and biological annotations of the samples have been declared to the CNIL (Comité National Informatique et Libertés, ie Data processing and Liberties National Committee).*<br>*Non leukemia controls were obtained from EFS BFC. EFS is a gouvernemental Agency collecting and delivering blood products, all procedures in use at EFS are defined by the Law. For samples aimed at reasearch use a personnal "informed consent" form is signed at the time of collection. This form was validated by the "Agence de la Biomedecine" the body which, in France, rules all type of samples of Human origin, on ethical and practical aspects, for research or clinical applications.*

Note that full information on the approval of the study protocol must also be provided in the manuscript.

Clinical data

Policy information about clinical studies

All manuscripts should comply with the ICMJE guidelines for publication of clinical research and a completed CONSORT checklist must be included with all submissions.

Clinical trial registration	*Provide the trial registration number from ClinicalTrials.gov or an equivalent agency.*
Study protocol	*Note where the full trial protocol can be accessed OR if not available, explain why.*
Data collection	*Describe the settings and locales of data collection, noting the time periods of recruitment and data collection.*
Outcomes	*Describe how you pre-defined primary and secondary outcome measures and how you assessed these measures.*

Dual use research of concern

Policy information about dual use research of concern

Hazards

Could the accidental, deliberate or reckless misuse of agents or technologies generated in the work, or the application of information presented in the manuscript, pose a threat to:

No	Yes	
☐	☐	Public health
☐	☐	National security
☐	☐	Crops and/or livestock
☐	☐	Ecosystems
☐	☐	Any other significant area

Experiments of concern

Does the work involve any of these experiments of concern:

No	Yes	
☐	☐	Demonstrate how to render a vaccine ineffective
☐	☐	Confer resistance to therapeutically useful antibiotics or antiviral agents
☐	☐	Enhance the virulence of a pathogen or render a nonpathogen virulent
☐	☐	Increase transmissibility of a pathogen
☐	☐	Alter the host range of a pathogen
☐	☐	Enable evasion of diagnostic/detection modalities
☐	☐	Enable the weaponization of a biological agent or toxin
☐	☐	Any other potentially harmful combination of experiments and agents

ChIP-seq

Data deposition

☐ Confirm that both raw and final processed data have been deposited in a public database such as GEO.

☐ Confirm that you have deposited or provided access to graph files (e.g. BED files) for the called peaks.

Data access links<br>*May remain private before publication.*	*For "Initial submission" or "Revised version" documents, provide reviewer access links. For your "Final submission" document, provide a link to the deposited data.*
Files in database submission	*Provide a list of all files available in the database submission.*
Genome browser session<br>(e.g. UCSC)	*Provide a link to an anonymized genome browser session for "Initial submission" and "Revised version" documents only, to enable peer review. Write "no longer applicable" for "Final submission" documents.*

Methodology

Replicates	*Describe the experimental replicates, specifying number, type and replicate agreement.*
Sequencing depth	*Describe the sequencing depth for each experiment, providing the total number of reads, uniquely mapped reads, length of reads and whether they were paired- or single-end.*

Antibodies	*Describe the antibodies used for the ChIP-seq experiments; as applicable, provide supplier name, catalog number, clone name, and lot number.*
Peak calling parameters	*Specify the command line program and parameters used for read mapping and peak calling, including the ChIP, control and index files used.*
Data quality	*Describe the methods used to ensure data quality in full detail, including how many peaks are at FDR 5% and above 5-fold enrichment.*
Software	*Describe the software used to collect and analyze the ChIP-seq data. For custom code that has been deposited into a community repository, provide accession details.*

Flow Cytometry

Plots

Confirm that:

- [x] The axis labels state the marker and fluorochrome used (e.g. CD4-FITC).
- [x] The axis scales are clearly visible. Include numbers along axes only for bottom left plot of group (a 'group' is an analysis of identical markers).
- [x] All plots are contour plots with outliers or pseudocolor plots.
- [x] A numerical value for number of cells or percentage (with statistics) is provided.

Methodology

Sample preparation	*AML cells in culture or from murine bone marrow and spleen were spun down and processed for staining.*
Instrument	*CytoFLEX flow cytometer (Beckman Coulter)*<br>*MACSQuant 10 or VYB (Miltenyi Biotec)*
Software	*FlowJo 10.4.2*
Cell population abundance	*No cell sorting was performed.*
Gating strategy	*Single cells were selected by FSC/SSC, FSC-H/FSC-A gates.*<br>*Human viable blasts from bone marrow and spleen of grafted mice were selected based on the expression of hCD45 and CD33. For all the experiments, viables AML cells were selected in the AnnexinV negative population. The gating strategy is provided in the Supplementary Information.*

- [x] Tick this box to confirm that a figure exemplifying the gating strategy is provided in the Supplementary Information.

Magnetic resonance imaging

Experimental design

Design type	*Indicate task or resting state; event-related or block design.*
Design specifications	*Specify the number of blocks, trials or experimental units per session and/or subject, and specify the length of each trial or block (if trials are blocked) and interval between trials.*
Behavioral performance measures	*State number and/or type of variables recorded (e.g. correct button press, response time) and what statistics were used to establish that the subjects were performing the task as expected (e.g. mean, range, and/or standard deviation across subjects).*

Acquisition

Imaging type(s)	*Specify: functional, structural, diffusion, perfusion.*
Field strength	*Specify in Tesla*
Sequence & imaging parameters	*Specify the pulse sequence type (gradient echo, spin echo, etc.), imaging type (EPI, spiral, etc.), field of view, matrix size, slice thickness, orientation and TE/TR/flip angle.*
Area of acquisition	*State whether a whole brain scan was used OR define the area of acquisition, describing how the region was determined.*

Diffusion MRI ☐ Used ☐ Not used

Preprocessing

Preprocessing software	*Provide detail on software version and revision number and on specific parameters (model/functions, brain extraction, segmentation, smoothing kernel size, etc.).*
Normalization	*If data were normalized/standardized, describe the approach(es): specify linear or non-linear and define image types used for transformation OR indicate that data were not normalized and explain rationale for lack of normalization.*
Normalization template	*Describe the template used for normalization/transformation, specifying subject space or group standardized space (e.g. original Talairach, MNI305, ICBM152) OR indicate that the data were not normalized.*
Noise and artifact removal	*Describe your procedure(s) for artifact and structured noise removal, specifying motion parameters, tissue signals and physiological signals (heart rate, respiration).*
Volume censoring	*Define your software and/or method and criteria for volume censoring, and state the extent of such censoring.*

Statistical modeling & inference

Model type and settings	*Specify type (mass univariate, multivariate, RSA, predictive, etc.) and describe essential details of the model at the first and second levels (e.g. fixed, random or mixed effects; drift or auto-correlation).*
Effect(s) tested	*Define precise effect in terms of the task or stimulus conditions instead of psychological concepts and indicate whether ANOVA or factorial designs were used.*

Specify type of analysis: ☐ Whole brain ☐ ROI-based ☐ Both

Statistic type for inference (See Eklund et al. 2016)	*Specify voxel-wise or cluster-wise and report all relevant parameters for cluster-wise methods.*
Correction	*Describe the type of correction and how it is obtained for multiple comparisons (e.g. FWE, FDR, permutation or Monte Carlo).*

Models & analysis

n/a	Involved in the study
☐	☐ Functional and/or effective connectivity
☐	☐ Graph analysis
☐	☐ Multivariate modeling or predictive analysis

Functional and/or effective connectivity	*Report the measures of dependence used and the model details (e.g. Pearson correlation, partial correlation, mutual information).*
Graph analysis	*Report the dependent variable and connectivity measure, specifying weighted graph or binarized graph, subject- or group-level, and the global and/or node summaries used (e.g. clustering coefficient, efficiency, etc.).*
Multivariate modeling and predictive analysis	*Specify independent variables, features extraction and dimension reduction, model, training and evaluation metrics.*
